# A Comparison of the Application of Four MEA-Based Neurotoxicity Screening Methods in Ni^2+^ Neurotoxicity Assessment

**DOI:** 10.3390/toxics14080719

**Published:** 2026-08-14

**Authors:** Chen Meng, Yang Lu, Yan Huang, Zhi-Gong Wang, Xiao-Ying Lü

**Affiliations:** 1 State Key Laboratory of Digital Medical Engineering, Southeast University, Nanjing 210096, China; vince.mengchen@hotmail.com (C.M.); luyang@tisshue.com (Y.L.); hylucky@seu.edu.cn (Y.H.); 2 Institute of RF- & OE-ICs, Southeast University, Nanjing 210096, China; zgwang@seu.edu.cn

**Keywords:** microelectrode array, neurotoxicity, spontaneous activity, evoked activity, electrical stimulation threshold, excitability

## Abstract

Microelectrode array (MEA) technology is widely employed in electrophysiological research and, in recent years, has increasingly been applied in the screening of neurotoxicity. In this study, we investigated the neurotoxic effects of different concentrations of Ni^2+^ by analyzing its influence on the mean firing and bursting rates of spontaneous activity, as well as the frequency of electrical-stimulation-evoked activity in the neocortical region in rat brain slices through MEA. Integrating our team’s prior findings on Ni^2+^’s influence on the electrical stimulation threshold of evoked activity, we compared the application effectiveness of the four MEA-based neurotoxicity screening methods in Ni^2+^ neurotoxicity research. Our results demonstrate that all of these methods can effectively assess the neurotoxic effects of Ni^2+^; however, they differ in certain aspects, such as the applicable neuronal networks and data-processing approaches. We also found that Ni^2+^ influences spontaneous and electrical-stimulation-evoked activity in the neocortical region in rat brain slices via distinct mechanisms.

## 1. Introduction

Neurotoxicity refers to the adverse effects of chemical substances on the structure or function of the nervous system, with a primary focus on functional alterations directly linked to the electrical excitability of neurons and neuronal networks [1,2]. Commonly used in vitro cytotoxicity assays are limited to detecting cell proliferation, alterations in cellular morphology and structure, and selected molecular biomarkers; they cannot capture changes in the electrical excitability of neurons and neuronal networks [3]. Consequently, these assays cannot fully reflect the neurotoxic effects induced by chemical substances. Currently, most neurotoxic effects can only be identified through time- and resource-intensive in vivo animal experiments, such as those assessing reflexes and ocular signs [4,5,6]. Therefore, establishing a reliable in vitro detection method capable of monitoring changes in the electrical excitability of neurons and neuronal networks, as well as integrating this method with conventional in vitro cytotoxicity assays, is critical for evaluating chemicals’ neurotoxic effects in the early stages of drug development.

Microelectrode array (MEA) technology enables real-time detection of the electrophysiological activity of neuronal networks composed of multiple neurons, thereby overcoming the limitations of single-cell electrophysiological detection methods such as patch-clamp techniques, which can only detect one neuron at a time. Consequently, not only has MEA been extensively utilized in basic electrophysiological research, but it is also increasingly applied in the investigation of chemical-substance-induced neurotoxicity. In recent years, several companies, such as Multichannel Systems, Axion Biosystems, and MaxWell Biosystems, have developed multi-well plates integrated with MEAs. These platforms retain the multi-channel recording capacity of traditional MEAs while enabling high-throughput screening. As a result, this emerging class of MEA-based platforms demonstrates considerable potential for large-scale neurotoxicity screening of pharmaceutical compounds [6,7,8].

The primary signals recorded by MEAs are extracellular potentials, and further insights can be gained by analyzing the spatial and temporal characteristics of the recorded activity. The most fundamental metrics typically assessed include the firing rate of action potentials (referred to as “spikes”) and the frequency of action potential clusters (referred to as “bursts”), which are composed of consecutive action potentials occurring within a short time interval [6]. Currently, MEA-based neurotoxicity assessment methods can be broadly categorized into two main types: those that monitor changes in spontaneous neuronal activity and those that evaluate alterations in evoked neuronal activity [6,9]. In the analysis of spontaneous activity changes, evaluating the mean firing rate of spikes and analyzing the mean bursting rate are two of the most fundamental and widely adopted approaches [6]. In the detection of electrical-stimulation-evoked activity, the reliability of this activity (i.e., the probability of generating evoked responses to stimulation) decreases significantly with an increase in the time since electrical stimulation. Typically, the reliability of evoked activity recorded beyond 50 ms post-stimulus drops below 50%. Consequently, analyses of electrically evoked activity generally focus on neuronal activity occurring within a short time window immediately following stimulation (usually less than 100 ms) [10]. Given the limited number of bursts that can occur within such a short time window, the bursting rate is generally not analyzed. Instead, the most commonly used approach is to evaluate the frequency characteristics of electrical-stimulation-evoked spikes [9,10,11,12]. However, the complexity of data processing in analyzing spike frequency evoked by electrical stimulation limits its utility in large-scale screening applications. To address this challenge, our research team developed a novel neurotoxicity assessment method in a previous study. This approach enables effective evaluation of the impact of chemicals on neuronal network excitability by analyzing changes in the electrical stimulation threshold required to evoke neuronal activity. Moreover, its simplified data-processing workflow enhances its suitability for future large-scale screening applications based on high-throughput MEA technologies [13].

Ni^2+^ is a common inhibitor of voltage-gated Ca^2+^ channels and NMDA receptors. Relevant studies have demonstrated that it suppresses neuronal excitability and exhibits neurotoxic effects [14,15,16,17]. In this study, the MEA technique was utilized to investigate the effects of varying Ni^2+^ concentrations on the mean firing and bursting rates of spontaneous neuronal activity, as well as the frequency of electrical-stimulation-evoked activity in the neocortex in rat brain slices. Furthermore, by integrating our team’s previous findings on Ni^2+^’s impact on the electrical stimulation threshold of evoked activity in the same brain region, we conducted a comparative evaluation to assess the efficacy of the four neurotoxicity assessment methods in detecting Ni^2+^-induced neurotoxicity. These results are expected to serve as a valuable reference for future research in this area.

## 2. Materials and Methods

### 2.1. Brain Slice Preparation

All animal procedures were approved by the Animal Ethics Committee of Southeast University, Jiangsu, China, and all methods were performed in accordance with their relevant guidelines and regulations. Our work also complies with the ARRIVE guidelines. A total of 30 specific-pathogen-free (SPF) Sprague–Dawley rats (14 days old; mixed sex; body weight: 20–35 g; Shanghai Laboratory Animal Research Center, Shanghai, China) were used in the experiments: 15 for spontaneous activity recording and 15 for evoked activity recording. The brains of the rats were rapidly explanted and rinsed with cold, oxygenated (95% O_2_, 5% CO_2_) artificial cerebrospinal fluid (ACSF) after the rats were euthanized via cervical dislocation. The composition of the ACSF was as follows: 125 mM NaCl, 3 mM KCl, 1.25 mM NaH_2_PO_4_, 1.6 mM CaCl_2_, 1.5 mM MgSO_4_, 26 mM NaHCO_3_, and 10 mM glucose. The isolated brain hemispheres were sectioned into coronal slices with a thickness of 300 μm using a tissue chopper (McIlwain, CLE, Loughborough, UK) and immediately transferred to oxygenated ACSF maintained at 32 °C [18,19]. After 30 min of incubation, a slice was quickly moved into the chamber of an MEA (60MEA200/30iR-Ti-pr-T, MCS, Reutlingen, Germany). The neocortical region in the slice was aligned with the electrodes at the center of the MEA chamber, and the slice was secured using a steel ring topped with nylon mesh. Subsequently, the MEA chamber was filled with 2 mL of oxygenated ACSF maintained at 32 °C, and the MEA was mounted onto the recording system (MEA2100, MCS, Reutlingen, Germany). The MEA chamber was continuously perfused with oxygenated ACSF at a rate of approximately 1 mL/min. The temperature of the ACSF in the MEA chamber was maintained at about 32 °C via a temperature controller (TC-02, MCS, Reutlingen, Germany). For each rat, one brain slice exhibiting spontaneous neocortical activity on more than 20% of functional MEA channels was selected for testing.

### 2.2. Recording and Analysis of Spontaneous Activity

After connecting the MEA with the brain slice to the recording system, we allowed the brain slice in the MEA chamber to rest for 45 min to recover from the stress induced by the preparation process. Subsequently, the first 45-min recording of spontaneous activity was performed. The perfusion solution was then replaced with oxygenated ACSF containing different concentrations (0, 50, 100, 200, or 500 μM) of NiCl_2_. Following a 20-min incubation period, a second 45-min recording of spontaneous activity from the same brain slice was obtained. Data acquired during the first and second recording sessions served as the control and experimental group data, respectively. During data analysis, the wave_clus (v3.03) MATLAB toolkit was executed in MATLAB 2020b to identify spikes recorded by all electrodes [20]. Customized MATLAB code was then applied to identify the bursts using the MaxInterval method [21,22]. Changes in the mean firing and bursting rates of spontaneous activity across all electrodes in the two recording sessions were analyzed to evaluate the neurotoxic effects of Ni^2+^ [6,21]. Data from an electrode were excluded from analysis if the mean firing rate in the control group was below 0.1 spikes/s [23].

### 2.3. Frequency Analysis of Electrical Stimulation Evoked Activity

#### 2.3.1. Evoked Activity Recording and Electrical Stimulation Parameter Configuration

Recording and electrical stimulation were all performed using the MEA2100 system. The recording sampling rate was 10 kHz. Before the recording, the MEA with the brain slice mounted on the MEA2100 system was allowed to rest for 45 min to enable the brain slice to recover from stress. One electrode near the center of the electrode array capable of recording spontaneous activity was designated as the stimulating electrode, while the remaining electrodes served as recording electrodes. Initial electrical stimulation and recording were performed to establish control group data. To investigate the effect of Ni^2+^ on evoked activity, after the initial stimulation, the perfusion solution was switched to oxygenated ACSF containing different concentrations (0, 50, 100, 200, or 500 μM) of NiCl_2_, and the brain slice was incubated in this solution for 20 min. Subsequently, electrical stimulation and recording were repeated on the same brain slice to obtain experimental group data.

**Figure 1:**
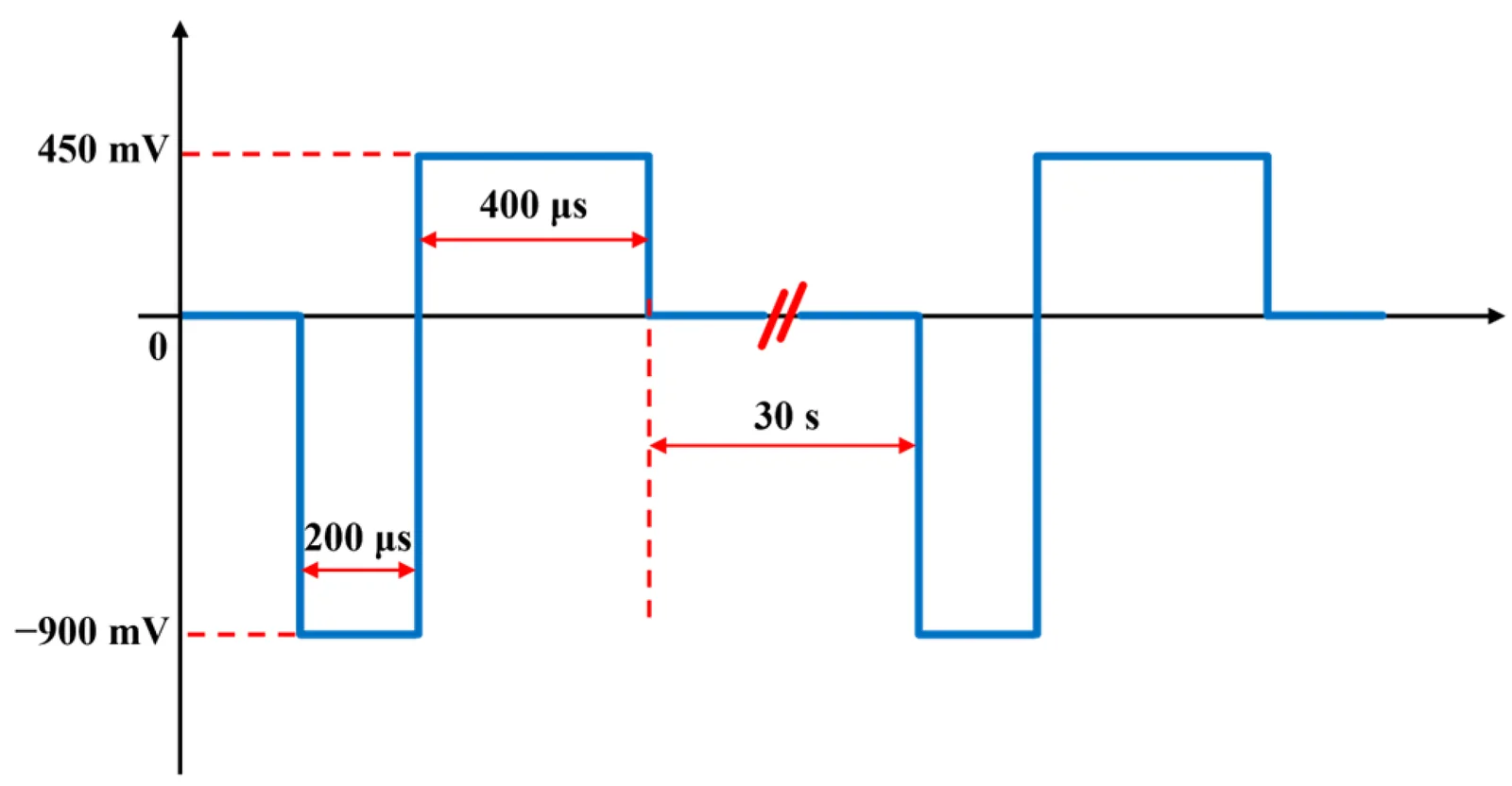
The waveform of electrical stimulation.

In the experiment, the waveform and frequency of stimulation were consistent with those used in our previous studies [13]. As the intensity of electrical stimulation increases, the number of electrodes capable of detecting evoked activity rises accordingly, thereby expanding the sample size obtained in each experiment. However, when the stimulation intensity exceeds 1000 mV, electrochemical reactions may occur at the electrode–tissue interface, potentially damaging the tissue cells [10]. Our previous research demonstrated that under conditions identical to those used in this study, an electrical stimulation intensity of 900 mV was sufficient to obtain enough electrodes to reliably detect evoked activity [13]. Therefore, in this experiment, the stimulation intensity was set to 900 mV to ensure we had a reliable number of responsive electrodes per trial while minimizing potential neuronal damage caused by excessive stimulation. The waveform of the electrical stimulation is shown in Figure 1. The electrical stimulation sequence used in each experiment consisted of 60 consecutive individual stimulations.

#### 2.3.2. Identification of Electrical-Stimulation-Evoked Activity

**Figure 2:**
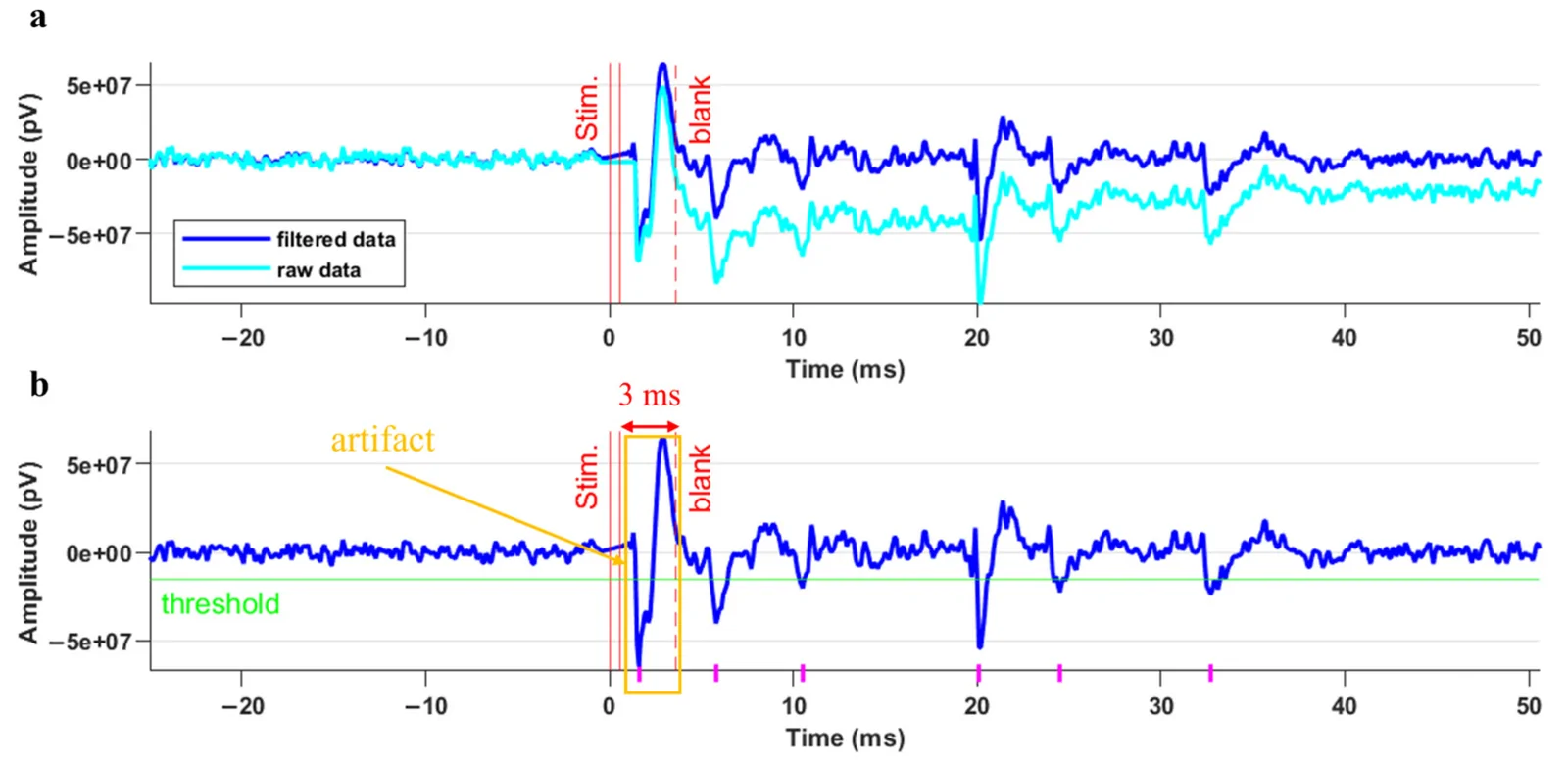
The response waveforms of neuronal networks in the neocortex in rat brain slices to a 900-mV electrical stimulus. (**a**) Representative waveforms of the raw signal (cyan) and the filtered signal (blue) recorded from the electrode following electrical stimulation in the experiment. (**b**) Spike detection result of the filtered signal (blue) in (**a**). The green line represents the threshold of spike detection. The magenta stubs on the time axis show the spike train of this channel obtained from the spike detection result. A prominent artifact occurred immediately following the stimulation (indicated by the orange box), which was erroneously identified as a spike (represented by the first short magenta line on the time axis). This detection error could be resolved by introducing a 3 ms blank after the stimulation.

The method for identifying electrical-stimulation-evoked activity was consistent with the approach used in our prior related research [13]. In brief, spikes were first detected using the wave_clus (v3.03) MATLAB toolkit based on the filtered data [20]. During analysis, we primarily focused on the spikes occurring within 50 ms post-stimulus. These spikes were predominantly generated by neurons directly activated by the electrical stimulation and their immediately connected downstream neurons [10]. We employed a 3 ms post-stimulus blank to eliminate artifacts’ influence on spike detection (Figure 2). In the experiment, the evoked spikes were identified on the basis of changes in spike density before and after electrical stimulation [13,24]. In the control group experiments, an electrode capable of detecting evoked activity in 50% of the trials within a stimulation sequence was considered to be capable of reliably detecting stable evoked activity and thus designated as an effective electrode.

#### 2.3.3. Frequency Analysis of Evoked Activity

**Figure 3:**
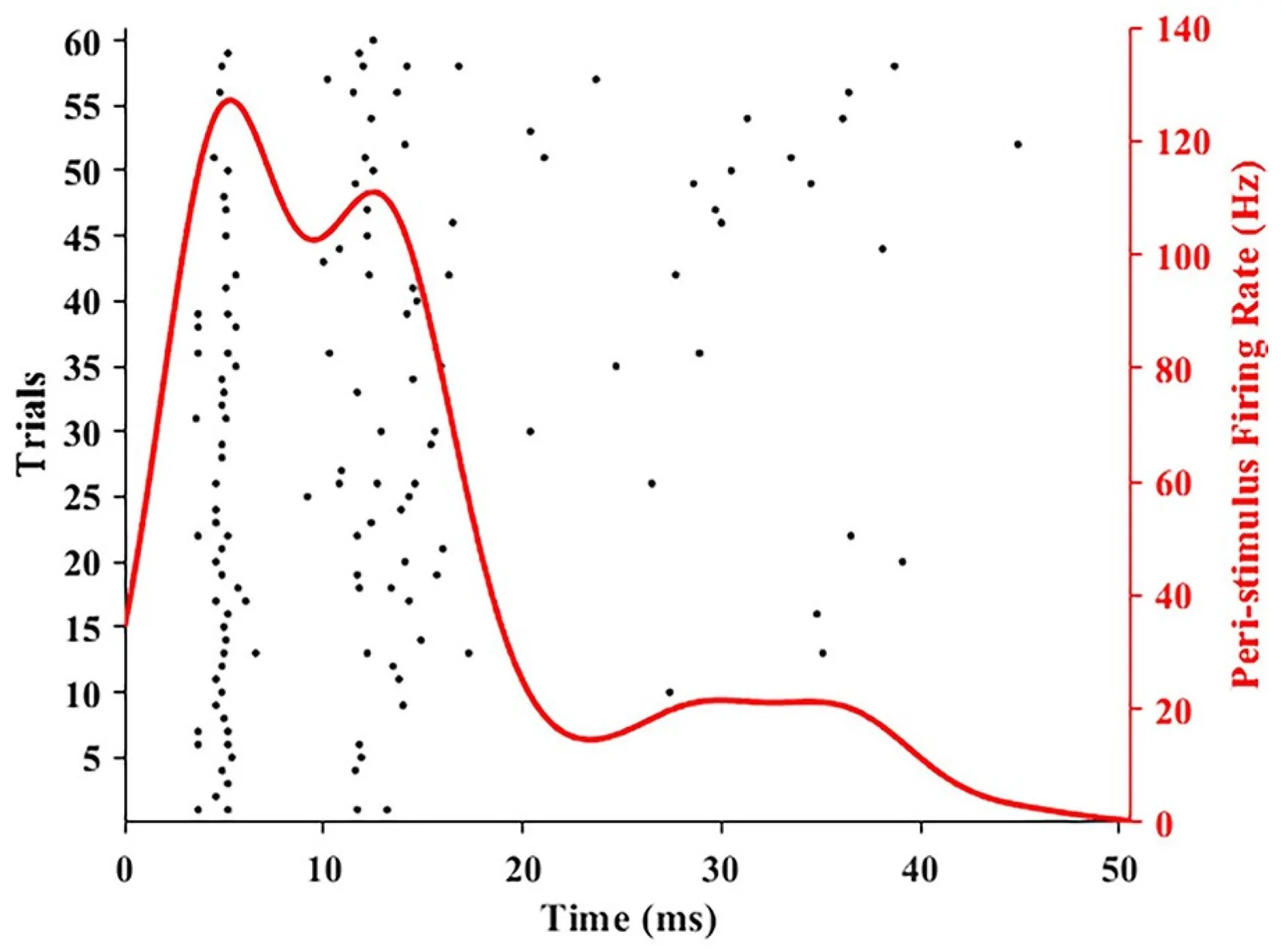
An example of the spike raster plot (black dots) for one electrode in the control group within 50 ms post-stimulus, along with the peri-stimulus spike firing rate (PSFR) curve (red) estimated via Gaussian kernel density estimation.

The peri-stimulus firing rate (PSFR) curves (Figure 3) for each effective electrode within 50 ms post-stimulus were computed using Gaussian kernel density estimation (kernel width = 3 ms) via customized MATLAB code [22,25]. The impact of Ni^2+^ on the neuronal network’s evoked activity was assessed by comparing the amplitude and timing of the first peak in the corresponding frequency curves from the same electrode in both the control and experimental groups. If no evoked activity was detected in the experimental group’s data for an electrode, the amplitude of the first peak of the PSFR of the electrode was set to 0. Given that peak timing cannot be determined under these conditions, data from this electrode were excluded from the statistical analysis of peak timing.

#### 2.3.4. Analysis of the Electrical Stimulation Threshold for Evoked Activity

The electrical stimulation threshold detection results for evoked activity reported herein were obtained from a prior independent experiment conducted by our research group. For details on the experimental procedures, please refer to our group’s previously published paper [13]. In brief, using the MEA2100 system, we delivered a series of electrical stimuli with intensities ranging incrementally from 100 mV to 1000 mV to the neocortical region of rat brain slices before and after exposure to different concentrations of Ni^2+^. The lowest stimulation voltage that stably elicited evoked activity, as recorded by each electrode, was defined as the electrical stimulation threshold for evoked activity at that electrode. Changes in this threshold before and after Ni^2+^ application were compared to evaluate the effect of Ni^2+^ on the excitability of the neuronal network.

### 2.4. Data Process and Analysis

The experimental results were analyzed using Origin 2025 software. Data normality was assessed using the Shapiro–Wilk test, and nonparametric tests were applied when normality assumptions were violated. The Kruskal–Wallis test and Conover–Iman post hoc test were performed via the Post-hoc Analysis for Nonparametric Tests application (v1.33) plugin in Origin 2025. For multiple comparisons, the Holm correction was applied to calculate adjusted *p* values. All statistical tests were two-tailed, and differences were deemed statistically significant when *p* < 0.05.

## 3. Results

### 3.1. The Effect of Ni^2+^ on the Mean Firing and Bursting Rates of Spontaneous Activity in Neuronal Networks

**Figure 4:**
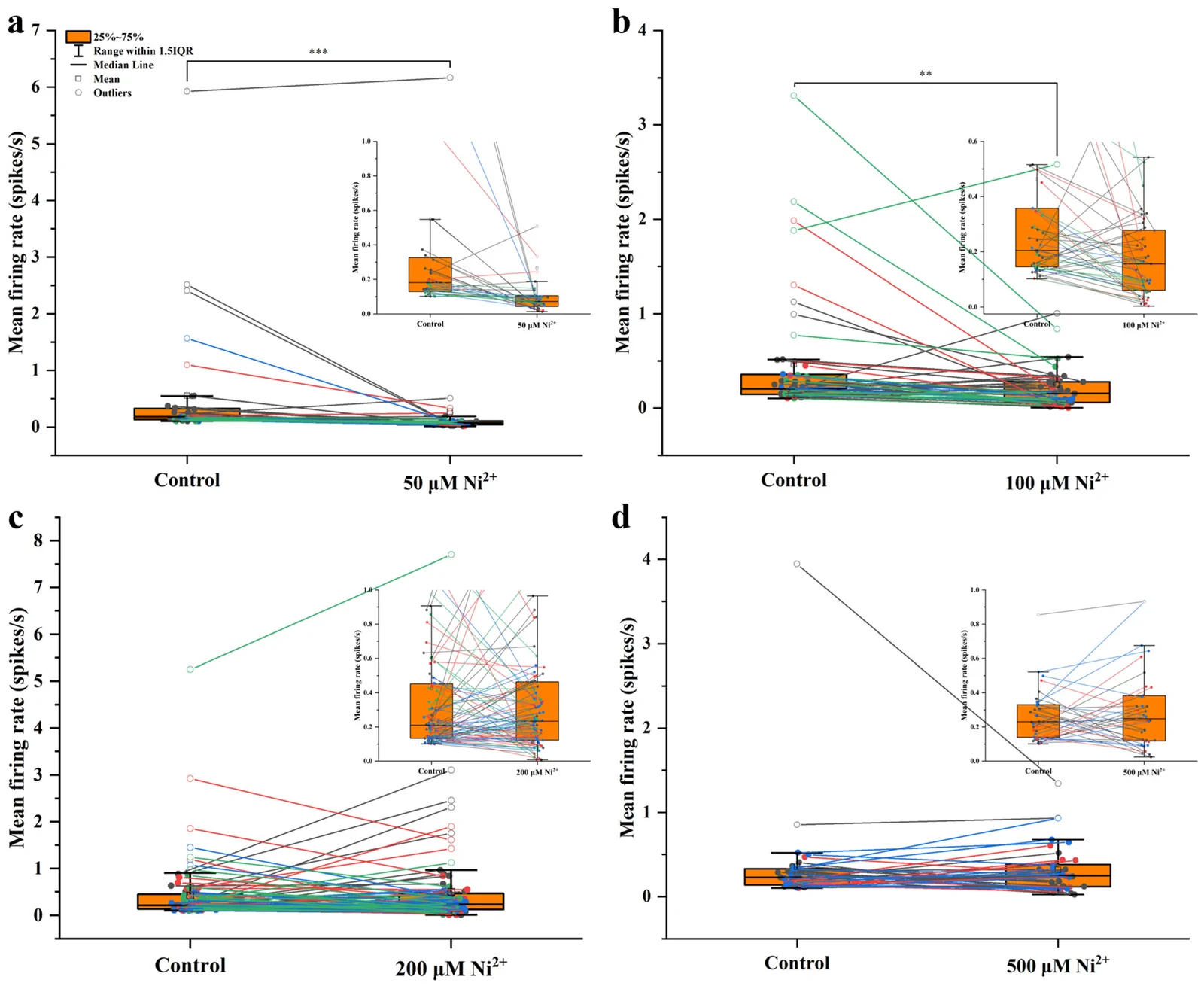
Changes in the mean firing rate of spontaneous activity before and after the application of Ni^2+^. The data points and lines in different colors correspond to different slices, and the insets provide a detailed view of the low-frequency range. ** *p* < 0.01, *** *p* < 0.001 compared with the control group. (**a**) Changes caused by 50 μM Ni^2+^ (*n* = 36, from 4 brain slices). *p* < 0.001. (**b**) Changes caused by 100 μM Ni^2+^ (*n* = 49, from 4 brain slices). *p* = 0.001. (**c**) Changes caused by 200 μM Ni^2+^ (*n* = 90, from 4 brain slices). *p* = 0.866. (**d**) Changes caused by 500 μM Ni^2+^ (*n* = 38, from 3 brain slices). *p* = 0.805.

**Figure 5:**
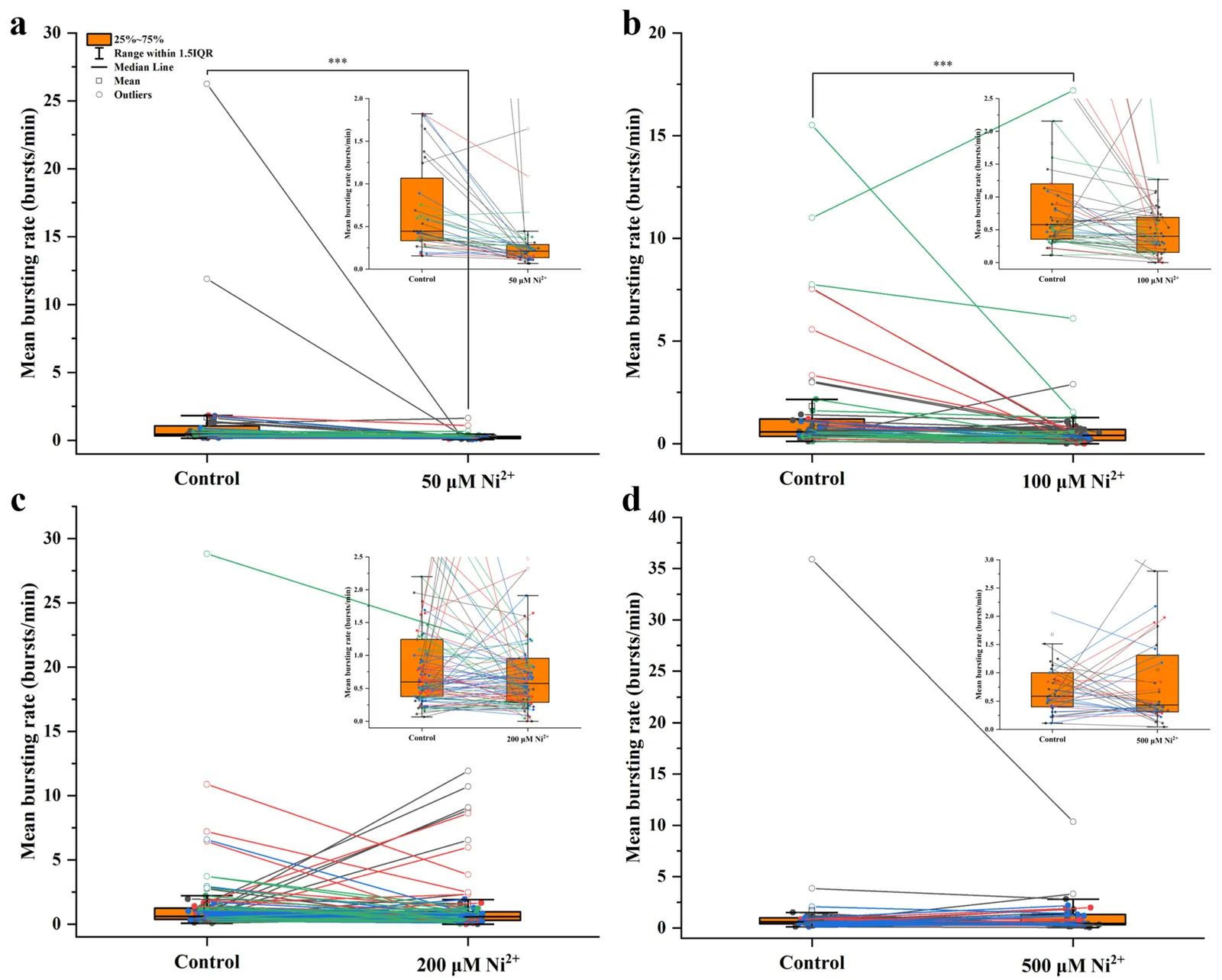
Changes in the mean bursting rate of spontaneous activity before and after the application of Ni^2+^. The data points and lines in different colors correspond to different slices, and the insets provide a detailed view of the low-frequency range. *** *p* < 0.001 compared with the control group. (**a**) Changes caused by 50 μM Ni^2+^ (*n* = 36, from 4 brain slices). *p* < 0.001. (**b**) Changes caused by 100 μM Ni^2+^ (*n* = 49, from 4 brain slices). *p* < 0.001. (**c**) Changes caused by 200 μM Ni^2+^ (*n* = 90, from 4 brain slices). *p* = 0.249. (**d**) Changes caused by 500 μM Ni^2+^ (*n* = 38, from 3 brain slices). *p* = 0.523.

In this study, two of the most widely used methods for analyzing spontaneous neuronal network activity—i.e., measuring mean firing rate and measuring mean bursting rate—were employed to assess the neurotoxic effects of different concentrations of Ni^2+^ [6]. The experimental data (Tables S1–S4) were subjected to statistical analysis using the paired-sample Wilcoxon signed-rank test, with the results presented in Figure 4 and Figure 5, respectively. Relative to the control group, exposure to 50 or 100 μM Ni^2+^ significantly reduced both the mean firing and bursting rates of spontaneous activity, indicating a substantial suppression of network excitability. This finding is consistent with the well-documented role of Ni^2+^ as an inhibitor of voltage-gated Ca^2+^ channels and NMDA receptors [13,14,15,16,17]. However, at Ni^2+^ concentrations of 200 or 500 μM, no statistically significant differences were detected in either the mean firing or bursting rates compared to the control group. These findings are consistent with our previous research, which demonstrated that 500 μM Ni^2+^ had no significant influence on these two activity characteristics [13]. In this study, the mean firing and bursting rates of spontaneous activity exhibited consistent trends before and after exposure to different concentrations of Ni^2+^. Importantly, the firing rate refers to the number of spikes generated within a defined time window, while the bursting rate primarily reflects the temporal organization of neuronal activity. These two metrics reflect distinct electrophysiological characteristics, and their responses under experimental conditions may not always exhibit consistency [17].

### 3.2. The Effect of Ni^2+^ on the Frequency of Electrical-Stimulation-Evoked Activity in Neuronal Networks

**Figure 6:**
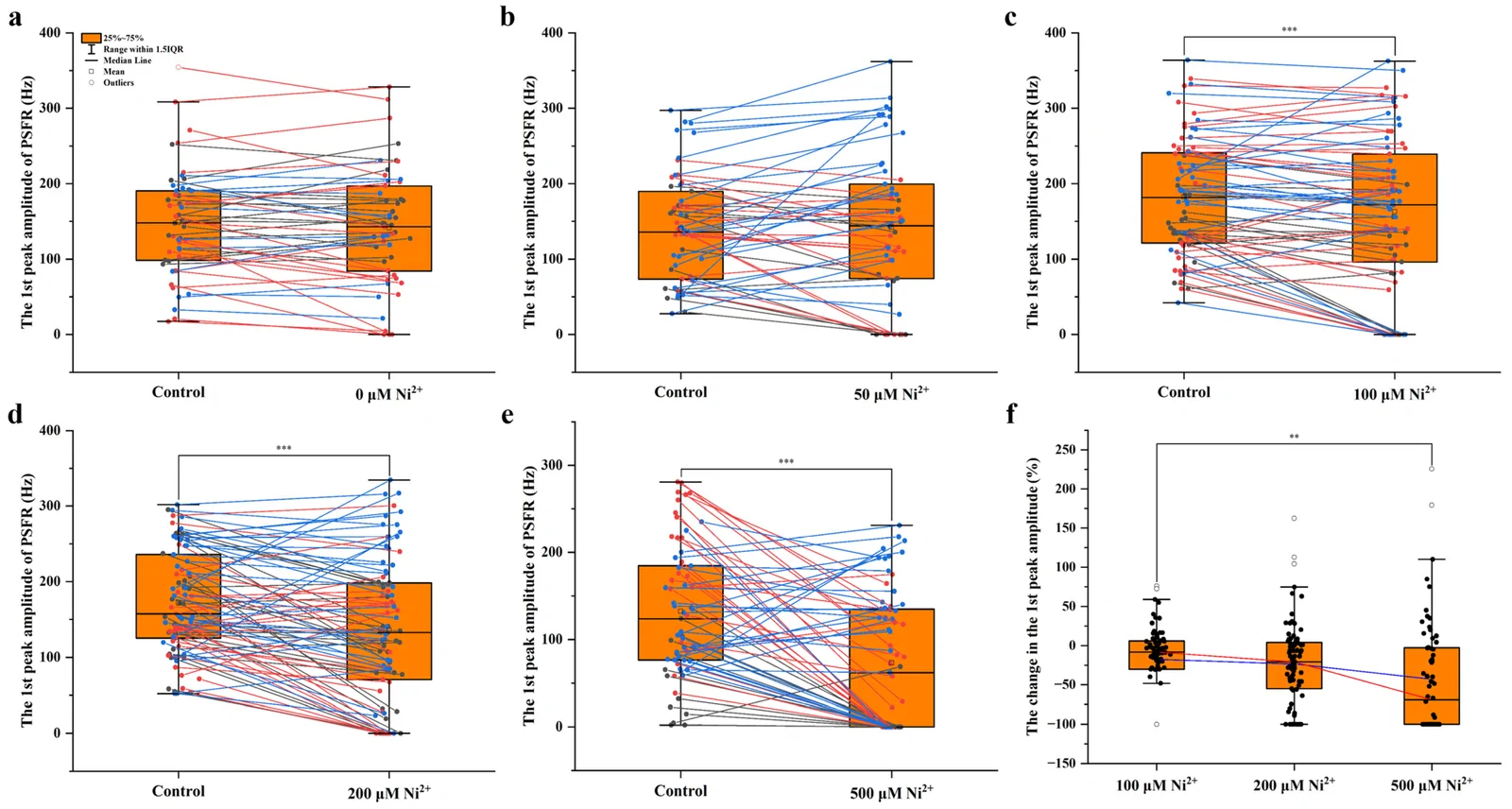
The influence of Ni^2+^ on the amplitude of the first PSFR peak. The data points and lines with different colors correspond to different slices. ** *p* < 0.01, *** *p* < 0.001 compared with the control group. (**a**) The comparison between the first PSFR peak amplitude of the control group and the Ni^2+^-free experimental group (*n* = 51, from 3 brain slices). *p* = 0.296. (**b**) The first PSFR peak amplitude before and after the application of 50 μM Ni^2+^ (*n* = 55, from 3 brain slices). *p* = 0.963. (**c**) The first PSFR peak amplitude before and after the application of 100 μM Ni^2+^ (*n* = 74, from 3 brain slices). *p* < 0.001. (**d**) The first PSFR peak amplitude before and after the application of 200 μM Ni^2+^ (*n* = 87, from 3 brain slices). *p* < 0.001. (**e**) The first PSFR peak amplitude before and after the application of 500 μM Ni^2+^ (*n* = 65, from 3 brain slices). *p* < 0.001. (**f**) The changes in the first PSFR peak amplitude caused by different concentrations of Ni^2+^; here, *n* = 74, *n* = 87, and *n* = 64 for the 100, 200 and 500 μM Ni^2+^ groups, respectively. *p* = 0.217, *p* = 0.005 and *p* = 0.063 for comparisons of the 100 vs. 200 μM Ni^2+^, 100 vs. 500 μM Ni^2+^ and 200 vs. 500 μM Ni^2+^ groups, respectively. The red and blue lines show the changes in medians and means, respectively.

In the analysis of the frequency of evoked activity induced by electrical stimulation, the neurotoxic effects of varying Ni^2+^ concentrations were evaluated by comparing the amplitude and timing of the first peak of the PSFR curve within 50 ms post-stimulus. Statistical analysis of the control and experimental group data was performed using the paired-sample Wilcoxon signed-rank test. The results (Tables S5–S9) demonstrated that the timing of the first peak of the PSFR was not significantly influenced by varying Ni^2+^ concentrations, whereas the amplitude of this peak decreased progressively with an increase in Ni^2+^ concentration (Figure 6). In the experiment, the first peaks of the PSFR in both the Ni^2+^-free experimental group and the experimental group exposed to 50 μM Ni^2+^ exhibited no significant differences compared to the control group, suggesting that neither the electrical stimulation itself nor the application of 50 μM Ni^2+^ exerted a significant effect (Figure 6a,b). In contrast, Ni^2+^ concentrations of 100, 200, or 500 μM all significantly reduced the amplitude of the first peak of the PSFR (Figure 6c–e). We further analyzed the percentage changes in PSFR peak amplitude (Table S10) induced by 100, 200, or 500 μM Ni^2+^ relative to the control group using the Kruskal–Wallis test followed by the Conover–Iman post hoc comparisons with Holm-adjusted *p* value; results are presented in Figure 6f. As the Ni^2+^ concentration increased, the reduction in the percentage changes in the first PSFR peak amplitude exhibited an increasing trend. These findings demonstrate that Ni^2+^ exerts an inhibitory effect on neuronal network activity evoked by electrical stimulation, with the inhibitory strength increasing in a concentration-dependent manner. This result is consistent with the results of our previous studies [13].

### 3.3. The Effect of Ni^2+^ on the Electrical Stimulation Threshold of Evoked Activity in Neuronal Networks

In our previous study, we examined changes in the electrical stimulation threshold of evoked activity in the neocortical region in rat brain slices before and after exposure to 50, 100, 200, or 500 μM Ni^2+^. The results demonstrate that Ni^2+^ exerts an inhibitory effect on the excitability of neuronal networks, with the inhibitory strength increasing in a concentration-dependent manner. Compared with the control group, the median of electrical stimulation threshold at the recording electrode increased by 14.3%, 20%, 36.7%, and 72.2% following exposure to 50, 100, 200, or 500 μM Ni^2+^, respectively [13].

## 4. Discussion

In this study, the effects of varying Ni^2+^ concentrations on the mean spontaneous firing and bursting rates and the frequency of electrical-stimulation-evoked activity in the neocortex in rat brain slices were assessed through the MEA technique. By integrating our team’s previous findings on the impact of Ni^2+^ on the electrical stimulation threshold of evoked activity in the same brain region, we conducted a comparative evaluation to assess the efficacy of these MEA-based neurotoxicity assessment methods [13]. The results of our experiments demonstrate that all four detection methods can effectively identify the neurotoxic effects of Ni^2+^ (Table 1 and Tables S11–S14). The assessment results regarding the mean firing and bursting rates of spontaneous activity consistently indicated that 50 or 100 μM Ni^2+^ significantly suppressed spontaneous activity in the neuronal network. However, at concentrations of 200 or 500 μM, no statistically significant differences were observed between the control and experimental groups, indicating a loss of inhibitory effect. In the analysis of electrical-stimulation-evoked activity, no significant difference in the first PSFR peak before and after the treatment of 50 μM Ni^2+^ (Figure 6b). However, exposure to 100, 200, or 500 μM Ni^2+^ resulted in a significant reduction in the first PSFR peak relative to the control group (Figure 6c–e), with median decreases of 8.23%, 20.88%, and 69.08%, respectively. These findings indicate that the inhibitory effect of Ni^2+^ on electrical-stimulation-evoked activity intensifies with an increasing concentration (Figure 6f), a finding that aligns with our previous observations regarding the elevation of electrical stimulation thresholds [13].

### 4.1. Comparative Analysis of the Four MEA-Based Neurotoxicity Screening Methods Used in This Study

In studies utilizing MEAs for screening the neurotoxicity of chemical substances, the most widely adopted methods involve assessing neurotoxic effects based on analyzing drug-induced alterations in the spontaneous activity of neuronal networks. However, these approaches have significant limitations. First, spontaneous activity demonstrates substantial variability, which undermines the stability and reproducibility of experimental data. Second, reliable analysis of spontaneous activity necessitates a relatively high baseline activity frequency within the neuronal network, thereby rendering these methods unsuitable for networks exhibiting inherently low levels of spontaneous activity [4,6,23]. Compared to analysis of spontaneous activity, evoked activity analysis offers superior data stability and does not depend on the baseline spontaneous activity frequency of neuronal networks. In electrophysiological research, a classic method for evaluating the effects of chemical substances on neuronal networks involves measuring changes in evoked activity frequency before and after compound application under a fixed electrical stimulation intensity. However, this approach necessitates manual determination of an optimal stimulation intensity capable of reliably eliciting a response [9,10]. Currently, this method is rarely utilized in large-scale neurotoxicity screening [6].

In 2013, our research group developed the Voltage Threshold Measurement Method (VTMM), a novel approach to detecting the electrical stimulation threshold of evoked activity through MEAs [26]. We previously employed the VTMM to investigate the effects of various chemical substances, such as acetylcholine and ethanol, on two types of neuronal networks—in vitro-cultured hippocampal neuronal networks and hippocampal slices—as well as PC12 quasi-neuronal networks, thereby validating the efficacy and applicability of this approach [26,27,28]. This method was also extended to assess the neurotoxicity of silver nanoparticles [29,30]. The electrical stimulation threshold detection method mentioned in this study integrates the key concept of the VTMM with the well-established technique of evoked activity identification commonly used in traditional electrical-stimulation-induced activity frequency analysis. Our prior research demonstrated that this evoked-activity-based electrical stimulation threshold detection method can sensitively detect the neurotoxic effects of Ni^2+^ [13]. Compared to the spontaneous activity detection methods, the electrical stimulation threshold detection method exhibits superior data stability. Furthermore, in contrast to the classic electrical stimulation frequency analysis method and the VTMM, it eliminates the need for manual adjustment of stimulation amplitude during the experiment, thereby improving its applicability in large-scale toxicity screening.

In MEA experiments, the complexity of data analysis directly impacts the efficiency of large-scale neurotoxicity screening applications. We assessed the data-analysis-related complexity of the four methods described in this study by evaluating the number of analytical steps required for each method (Table 2). Among these, the spontaneous activity assessment methods demonstrated relatively low data-processing complexity. Following spike detection in the raw data, the mean firing and bursting rates of spontaneous activity can be directly calculated. After datasets with abnormally low average spike frequencies are excluded, the remaining data can be subjected to statistical analysis [23]. In the analysis of electrical-stimulation-evoked activity, an additional analytical step is required after spike detection to identify evoked responses, in contrast to the simpler processing involved in spontaneous activity analysis. The electrical stimulation threshold for evoked activity can be directly determined by analyzing stimulation intensities that consistently elicit evoked responses following this step, after which statistical analysis is performed on the collected data [13]. Regarding the analysis of evoked activity frequency, once evoked activity identification is completed, the PSFR following stimulation must first be calculated using kernel density estimation methods, such as Gaussian kernel density estimation. Subsequently, statistical analysis can be carried out on the processed data. Furthermore, during PSFR calculation, the kernel density estimation analysis window (kernel width) must be manually adjusted to optimize analytical performance. Consequently, the frequency analysis method for electrical-stimulation-evoked activity represents the most complex procedure among the four evaluated methods [22,25]. Additionally, the experimental durations vary across the four methods (Table 2). Notably, detection of the electrical stimulation threshold for evoked activity requires a significantly longer experiment relative to the other three methods. Prolonged experiments may affect the electrophysiological properties of acute tissue preparations, such as brain slices, thereby potentially confounding the observed effects and warranting careful consideration in data interpretation. Consequently, this method is more appropriately applied to in vitro cultured neuronal networks on MEAs.

### 4.2. Mechanistic Analysis of the Impact of Different Concentrations of Ni^2+^ on Spontaneous and Electrical-Stimulation-Evoked Activity in Neuronal Networks

In our experiments, we observed distinct inhibitory effects of Ni^2+^ on neuronal networks in the neocortical region in rat brain slices between spontaneous activity recordings and electrical-stimulation-evoked activity measurements (Table 1). These differences suggest that the two major detection methods may reflect distinct electrophysiological properties.

**Figure 7:**
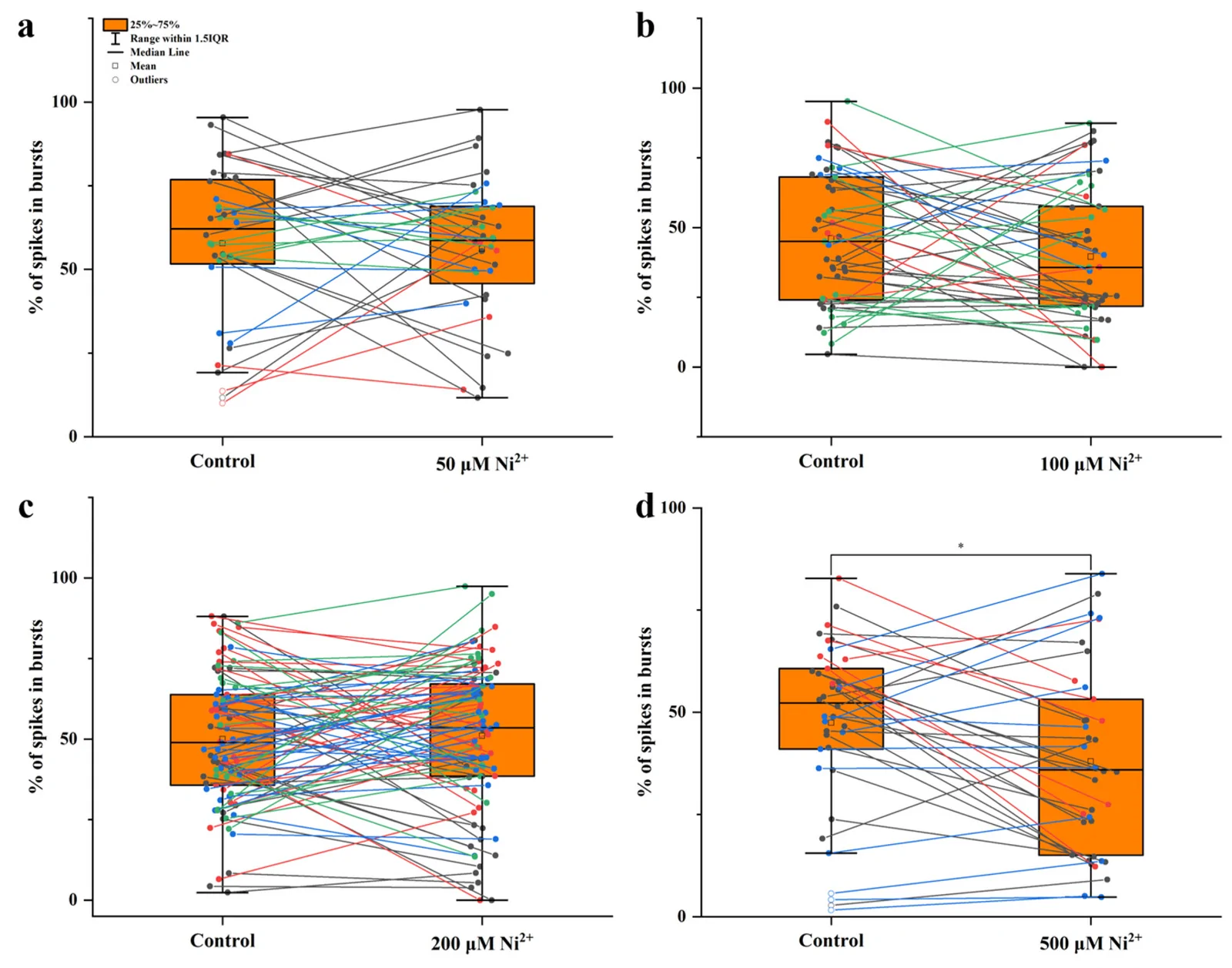
Changes in the percentage of spikes in bursts of spontaneous activity before and after the application of Ni^2+^. The data points and lines with different colors correspond to different slices. * *p* < 0.05 compared with the control group. (**a**) Changes caused by 50 μM Ni^2+^ (*n* = 36, from 4 brain slices). *p* = 0.689. (**b**) Changes caused by 100 μM Ni^2+^ (*n* = 49, from 4 brain slices). *p* = 0.105. (**c**) Changes caused by 200 μM Ni^2+^ (*n* = 90, from 4 brain slices). *p* = 0.483. (**d**) Changes caused by 500 μM Ni^2+^ (*n* = 38, from 3 brain slices). *p* = 0.012.

The spontaneous activity of neurons constitutes the self-generated electrical potential fluctuations that occur in the absence of external stimulation under stable environmental conditions. Research has demonstrated that such spontaneous activity persists even when synaptic transmission within the neural network is fully blocked [31,32]. Cohen et al. demonstrated that in the hippocampal CA3 pyramidal neuron network, blocking the synaptic excitatory transmission mediated by AMPA and NMDA receptors with the respective excitatory neurotransmitter receptor antagonists NBQX and D-AP5 caused the spontaneous activity frequency to decrease to 77 ± 35% of the control level. Further application of the inhibitory neurotransmitter receptor antagonist picrotoxin to block GABA receptor-mediated synaptic inhibition resulted in a significant increase in the spontaneous activity frequency to 177 ± 71% of the control level under conditions where both excitatory and inhibitory synaptic signals were negated. These findings indicate that inhibitory neurons exert a strong suppressive influence on spontaneous activity within the CA3 pyramidal neuronal network, and their regulatory effect on spontaneous activity frequency is more pronounced than that of excitatory neurons [31]. We propose that the phenomenon observed in this study—the lack of an inhibitory effect of 200 or 500 μM Ni^2+^ on spontaneous neuronal network activity—is most plausibly explained by a mechanism similar to that reported in the aforementioned CA3 study. Both findings appear to be closely associated with the suppression of synaptic inhibitory signalling. In this study, 50 or 100 μM Ni^2+^ primarily reduced network excitability by blocking T-type Ca^2+^ channels—particularly the Cav3.2 subtype—on the neuronal surface, whereas Ni^2+^’s effect on inhibitory neurons may remain minimal under these experimental conditions. Consequently, we observed a decrease in the spontaneous activity of neuronal networks (relative to the control group) in the neocortical region [14,15,16]. Notably, 200 or 500 μM Ni^2+^ inhibits synaptic excitatory transmission in neuronal networks by blocking NMDA receptors, consequently disrupting the propagation of signals that excite inhibitory neurons [33,34,35]. Furthermore, an excessive quantity of Ni^2+^ can impair the activity of inhibitory neurons by blocking multiple types of voltage-gated Ca^2+^ channels, ultimately leading to a decrease in the synaptic inhibitory signals they produce [16,17]. At this point, the blockade of synaptic inhibitory signals could result in a partial recovery of spontaneous activity in neuronal networks treated with 200 or 500 μM Ni^2+^ relative to those exposed to lower concentrations of Ni^2+^. However, in contrast to the synaptic signal blockers employed in the aforementioned CA3 study, 200 or 500 μM Ni^2+^ not only blocked both excitatory and inhibitory synaptic signals but also reduced network excitability by inhibiting voltage-gated Ca^2+^ channels. Consequently, the spontaneous activity frequency of the neuronal network did not significantly exceed that of the control group. Given that burst firing is tightly coupled with synaptic communication within neuronal networks, we further quantified the proportion of spikes occurring within bursts (the % of spikes in bursts) in spontaneous neocortical network activity to assess Ni^2+^-induced alterations in synaptic transmission [36]. Statistical analysis revealed that 500 μM Ni^2+^ produced a significant reduction in the % of spikes in bursts (Figure 7, Tables S15–S18). This shift reflects a more dispersed temporal distribution of spikes across the network, a finding consistent with our prior observations [13]. This disruption in burst architecture provides convergent functional evidence supporting the hypothesis that high-dose Ni^2+^ impairs synaptic transmission, thereby lending mechanistic plausibility to our proposed theoretical framework [36].

The evoked activity of neurons constitutes the evoked responses generated by the nervous system in response to various external stimuli [37]. Wagenaar et al. demonstrated that electrical stimulation in MEA experiments consistently evokes stable responses within 50 ms after a stimulus. Moreover, by analyzing how these responses were modulated following the application of glutamatergic synaptic transmission inhibitors and sodium channel blockers, their study confirmed that the evoked activity primarily originates from neurons directly activated by the electrical stimulus and their functionally connected counterparts [10]. Therefore, the characteristics of evoked activity observed in the MEA experiments described in this study reflect the excitability of the neuronal network spanning between the stimulating and detecting electrodes, with the majority of synaptic connections among these neurons being excitatory in nature. In this experiment and previous studies, we measured both the frequency of evoked activity occurring within 50 ms after electrical stimulation under varying concentrations of Ni^2+^ and the threshold of electrical stimulation required to elicit such activities [13]. The results consistently demonstrated that Ni^2+^’s inhibitory effect on electrical-stimulation-induced neuronal activity intensifies with an increase in concentration. These findings are also in line with the established role of Ni^2+^ as a blocker of voltage-gated Ca^2+^ channels and NMDA receptors [14,15,16,17,33,34,35].

**Figure 8:**
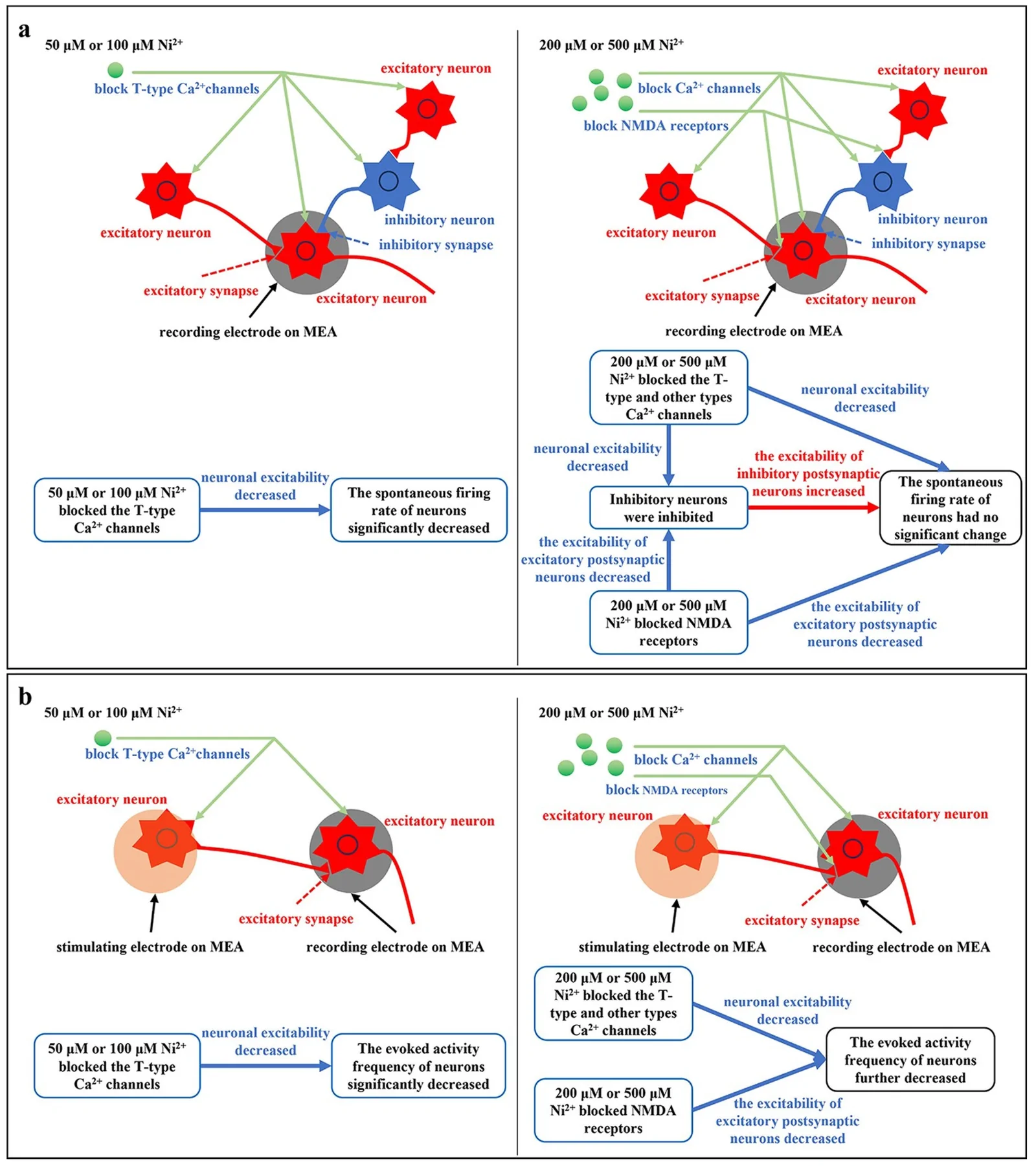
Hypothesized mechanism underlying the differential effects of Ni^2+^ on spontaneous and evoked activity, as revealed by multi-electrode array (MEA) recordings. (**a**) The effect of varying concentrations of Ni^2+^ on spontaneous activity reflects the combined modulation of Ni^2+^ on both excitatory and inhibitory neurons; (**b**) The effect of Ni^2+^ on evoked activity primarily reflects its modulation on excitatory neurons.

In summary, the alterations in spontaneous activity detected by MEA in response to varying concentrations of Ni^2+^ observed in this study may reflect the combined effects of Ni^2+^ on neuronal excitability as well as both excitatory and inhibitory synaptic connections within the neuronal network. In contrast, changes in evoked activity are predominantly influenced by neuronal excitability and excitatory synaptic transmission. Consequently, the impact of Ni^2+^ on spontaneous and evoked activity differs. A potential underlying mechanism is illustrated in Figure 8.

However, given the relatively small slice sample size in this study, additional experiments are needed to further validate the observed alteration in spontaneous activity. Moreover, our previous work has demonstrated that Ni^2+^ concentrations ≥200 μM exert cytotoxic effects, and prolonged exposure (≥24 h) to such doses significantly compromises neuronal viability and impairs signal transduction in primary rat neocortical neuronal cultures [38]. The non-specific effects of high doses of Ni^2+^ on the viability of the slice must be carefully considered, and the phenomena observed herein are detectable only in acute brain slice preparations under short-term experimental conditions. Furthermore, MEA-recorded data alone cannot reliably identify excitatory and inhibitory neurons within neuronal networks. Therefore, the proposed theoretical interpretation requires further experimental verification. In future studies, quantitative assessment of inhibitory neuron activity—through the application of specific receptor antagonists and complementary techniques such as patch-clamp recording, immunocytochemistry and Ca^2+^ imaging—will be critical for elucidating the underlying mechanisms by which Ni^2+^ modulates spontaneous activity in neuronal networks.

In this comparative study, although the neurotoxicity-screening method based on electrical stimulation thresholds offers advantages such as superior data stability and demonstrates considerable potential for large-scale neurotoxicity screening applications, it remains a relatively novel approach and has so far only been applied to assessing the neurotoxic effects of varying Ni^2+^ concentrations on rat brain slices. Further studies employing a broader range of materials and drugs across diverse neuronal network models are essential to further validate and refine this method. Notably, electrical stimulation threshold measurement is a network-level assessment that does not directly reflect changes in intrinsic neuronal properties, such as resting membrane potential, input resistance, or intrinsic firing characteristics. Rather than representing a genuine reduction in neuronal excitability, the observed increase in stimulation threshold may result from alterations in neuronal network connectivity or extracellular coupling effects. To further elucidate the mechanistic relationships among single-neuron electrophysiological properties, electrical stimulation thresholds, and other network-level functional metrics, studies could integrate MEA technology with single-cell potential recording techniques such as patch-clamp and voltage-sensitive dye imaging.

## 5. Conclusions

We utilized MEAs to investigate the effects of different concentrations of Ni^2+^ on three electrophysiological parameters in the neocortex region in rat brain slices: the mean spontaneous firing rate, the mean spontaneous bursting rate, and the frequency of electrical-stimulation-evoked activity. In conjunction with our previous findings on the impact of Ni^2+^ on the stimulation threshold for evoked activity, a comparative analysis was performed to evaluate the effectiveness of these four MEA-based electrophysiological methods in Ni^2+^ neurotoxicity screening. Additionally, the underlying mechanisms by which different Ni^2+^ concentrations affect both spontaneous and electrical-stimulation-evoked neuronal network activities were explored. All four methods demonstrated sensitivity to Ni^2+^-induced neurotoxicity. Notably, the novel electrical stimulation threshold detection method we have proposed shows significant potential for application in large-scale neurotoxicity screening. This work provides valuable insights useful for the practical implementation of MEA-based neurotoxicity screening technologies.

## Figures and Tables

**Table 1:** Neurotoxicity assessment outcomes regarding Ni^2+^ as determined via the four MEA experimental methods in this study.

Tested Features	Percentage Change in Median Values Across Experimental Groups Relative to the Control Groups			
	50 μM Ni^2+^	100 μM Ni^2+^	200 μM Ni^2+^	500 μM Ni^2+^
The mean spontaneous firing rate	−55.82	−49.11	NS *	NS *
The mean spontaneous bursting rate	−52.48	−33.33	NS *	NS *
The amplitude of the first PFSR peak	NS *	−8.23	−20.88	−69.08
The electrical stimulation threshold of evoked activity	+14.29	+20.00	+36.67	+72.22

* NS denotes the absence of statistically significant differences between the control and experimental group data.

**Table 2:** Comparison of the main characteristics of the MEA-based methods for Ni^2+^ neurotoxicity screening used in this study.

Method	Applicability	Data Analysis Procedures	Experiment Duration	Others
Assessment of the mean spontaneous firing rate	Not suitable for neuronal networks with low spontaneous activity frequencies Suitable for large-scale neurotoxicity-screening scenarios	(1) Detection of spikes (2) Calculation of spike frequencies (3) Statistical analysis of the data	About 3 h	The data exhibit high variability, necessitating a large sample. Data analysis requires commercial software or custom programming.
Assessment of the mean spontaneous bursting rate	Not suitable for neuronal networks with low spontaneous activity frequencies Suitable for large-scale neurotoxicity-screening scenarios	(1) Detection of spikes (2) Identification of burst activity and calculation of burst frequencies (3) Statistical analysis of the data	About 3 h	The data exhibit high variability, necessitating a large sample. The data simultaneously capture the quantity and pattern of spontaneous activity. Data analysis requires commercial software or custom programming.
Assessment of the frequencies of electrical-stimulation-evoked activity	Suitable for neuronal networks with low spontaneous activity frequencies Not suitable for large-scale neurotoxicity-screening scenarios	(1) Detection of spikes (2) Identification of evoked activity (3) PSFR calculation via kernel density estimation and identification of the PSFR peak (4) Statistical analysis of the data	About 2 h	Manual selection of an appropriate electrical stimulation intensity is required. Manual adjustment of kernel width is required to optimize the performance. Data analysis requires commercial software or custom programming.
Assessment of the electrical stimulation threshold for evoked activity	Suitable for neuronal networks with low spontaneous activity frequencies Suitable for large-scale neurotoxicity-screening scenarios	(1) Detection of spikes (2) Identification of evoked activity (3) Determination of the electrical stimulation threshold for evoked activity (4) Statistical analysis of the data	About 5 h	More appropriate for neuronal networks cultured in vitro on MEAs Currently, no commercial software is available for the assessment; therefore, data analysis must be conducted through custom programming.

## Data Availability

The data presented in this study are available within the article and its Supplementary Materials. Additional information and data are available from the corresponding author upon request.

## Supplementary Materials

The following supporting information can be downloaded at: https://www.mdpi.com/article/10.3390/toxics14080719/s1, Table S1: The spontaneous mean firing rate and mean bursting rate before and after treatment with 50 μM Ni^2+^; Table S2: The spontaneous mean firing rate and mean bursting rate before and after treatment with 100 μM Ni^2+^; Table S3: The spontaneous mean firing rate and mean bursting rate before and after treatment with 200 μM Ni^2+^; Table S4: The spontaneous mean firing rate and mean bursting rate before and after treatment with 500 μM Ni^2+^; Table S5: The amplitude and timing of the first peak in the peri-stimulus firing rate within 50 ms post-stimulus for control and Ni^2+^-free experimental groups; Table S6: The amplitude and timing of the first peak in the peri-stimulus firing rate within 50 ms post-stimulus for control groups and experimental groups treated with 50 μM Ni^2+^; Table S7: The amplitude and timing of the first peak in the peri-stimulus firing rate within 50 ms post-stimulus for control groups and experimental groups treated with 100 μM Ni^2+^; Table S8: The amplitude and timing of the first peak in the peri-stimulus firing rate within 50 ms post-stimulus for control groups and experimental groups treated with 200 μM Ni^2+^; Table S9: The amplitude and timing of the first peak in the peri-stimulus firing rate within 50 ms post-stimulus for control groups and experimental groups treated with 500 μM Ni^2+^; Table S10: The percentage changes in the amplitude of the first peak in the peri-stimulus firing rate within 50 ms post-stimulus induced by 100, 200, or 500 μM Ni^2+^; Table S11: Percentage change of the mean spontaneous firing rate across experimental groups relative to the control groups; Table S12: Percentage change of the mean spontaneous bursting rate across experimental groups relative to the control groups; Table S13: Percentage change of the amplitude of the first PFSR peak across experimental groups relative to the control groups; Table S14: Percentage change of the amplitude of the electrical stimulation threshold of evoked activity across experimental groups relative to the control groups; Table S15: The percentage of spikes in bursts of spontaneous activity for control groups and experimental groups treated with 50 μM Ni^2+^; Table S16: The percentage of spikes in bursts of spontaneous activity for control groups and experimental groups treated with 100 μM Ni^2+^; Table S17: The percentage of spikes in bursts of spontaneous activity for control groups and experimental groups treated with 200 μM Ni^2+^; Table S18. The percentage of spikes in bursts of spontaneous activity for control groups and experimental groups treated with 500 μM Ni^2+^.
