# Supplementary material for: A Comparison of the Application of Four MEA-Based Neurotoxicity Screening Methods in Ni2+ Neurotoxicity Assessment
Source: Toxics. 2026 Aug 14;14(8):719. doi: 10.3390/toxics14080719 (PMC13517805; doi:10.3390/toxics14080719)
Supplement: Supplementary file 1 [file toxics-14-00719-s001.zip › toxics-4469321-supplementary.pdf]

Supplementary Material

A comparison on the application of four MEA-based neurotoxicity screening methods in Ni^{2+} neurotoxicity assessment

Chen Meng[1], Yang Lu[1], Yan Huang[1], Zhi-Gong Wang[2], Xiao-Ying Lü[1,*]
1 State Key Laboratory of Digital Medical Engineering, Southeast University, Nanjing 210096, China
2 Institute of RF- & OE-ICs, Southeast University, Nanjing 210096, China
* Corresponding author.
E-mail address: luxy@seu.edu.cn (Xiao-Ying Lü).

Index

Table S1. The spontaneous mean firing rate and mean bursting rate before and after treatment with 50 μM Ni^{2+}. 4

Table S2. The spontaneous mean firing rate and mean bursting rate before and after treatment with 100 μM Ni^{2+}. 5

Table S3. The spontaneous mean firing rate and mean bursting rate before and after treatment with 200 μM Ni^{2+}. 7

Table S4. The spontaneous mean firing rate and mean bursting rate before and after treatment with 500 μM Ni^{2+}. 10

Table S5. The amplitude and timing of the first peak in the peri-stimulus firing rate within 50 ms post-stimulus for control and Ni^{2+}-free experimental groups. 11

Table S6. The amplitude and timing of the first peak in the peri-stimulus firing rate within 50 ms post-stimulus for control groups and experimental groups treated with 50 μM Ni^{2+}. 13

Table S7. The amplitude and timing of the first peak in the peri-stimulus firing rate within 50 ms post-stimulus for control groups and experimental groups treated with 100 μM Ni^{2+}. 15

Table S8. The amplitude and timing of the first peak in the peri-stimulus firing rate within 50 ms post-stimulus for control groups and experimental groups treated with 200 μM Ni^{2+}. 17

Table S9. The amplitude and timing of the first peak in the peri-stimulus firing rate within 50 ms post-stimulus for control groups and experimental groups treated with 500 μM Ni^{2+}. 20

Table S10. The percentage changes in the amplitude of the first peak in the peri-stimulus firing rate within 50 ms post-stimulus induced by 100, 200, or 500 μM Ni^{2+}. 22

Table S11. Percentage change of the mean spontaneous firing rate across experimental groups relative to the control groups. 25

Table S12. Percentage change of the mean spontaneous bursting rate across experimental groups relative to the control groups...25

Table S13. Percentage change of the amplitude of the first PFSR peak across experimental groups relative to the control groups...25

Table S14. Percentage change of the amplitude of the electrical stimulation threshold of evoked activity across experimental groups relative to the control groups.............25

Table S15. The percentage of spikes in bursts of spontaneous activity for control groups and experimental groups treated with 50 μM Ni^{2+}. 26

Table S16. The percentage of spikes in bursts of spontaneous activity for control groups and experimental groups treated with 100 μM Ni^{2+}. 27

Table S17. The percentage of spikes in bursts of spontaneous activity for control groups and experimental groups treated with 200 μM Ni^{2+}. 29

Table S18. The percentage of spikes in bursts of spontaneous activity for control groups and experimental groups treated with 500 μM Ni^{2+}. 32

Table S1. The spontaneous mean firing rate and mean bursting rate before and after treatment with 50 μM Ni^{2+}.

No.	Mean firing rate - control (spikes/s)	Mean bursting rate - control (bursts/min)	Mean firing rate - 50 μM Ni^{2+} (spikes/s)	Mean bursting rate - 50 μM Ni^{2+} (bursts/min)	Slice No.
1	0.339	0.444	0.029	0.089	1
2	0.114	0.333	0.089	0.200	1
3	2.516	26.244	0.146	0.444	1
4	2.409	11.889	0.043	0.067	1
5	0.143	0.689	0.073	0.244	1
6	5.927	1.800	6.169	0.289	1
7	0.200	0.333	0.059	0.311	1
8	0.263	0.267	0.013	0.111	1
9	0.242	0.156	0.020	0.111	1
10	0.548	1.644	0.031	0.067	1
11	0.144	0.267	0.138	0.178	1
12	0.373	0.444	0.054	0.133	1
13	0.547	1.378	0.043	0.289	1
14	0.314	1.311	0.048	0.133	1
15	0.149	0.422	0.187	0.178	1
16	0.102	0.356	0.097	0.244	1
17	0.234	1.244	0.509	1.644	1
18	0.255	0.533	0.021	0.111	1
19	0.183	0.289	0.020	0.156	2
20	0.198	0.378	0.245	0.111	2
21	0.122	0.178	0.086	0.133	2
22	1.099	1.822	0.332	1.089	2
23	1.564	1.800	0.071	0.133	3
24	0.183	0.578	0.049	0.222	3
25	0.122	0.200	0.038	0.156	3
26	0.134	0.422	0.088	0.111	3
27	0.147	0.444	0.100	0.267	3
28	0.180	0.889	0.034	0.200	3
29	0.102	0.178	0.100	0.222	3
30	0.125	0.622	0.063	0.222	4
31	0.174	0.622	0.148	0.667	4
32	0.162	0.378	0.062	0.244	4
33	0.104	0.267	0.111	0.289	4
34	0.104	0.356	0.095	0.378	4
35	0.142	0.756	0.073	0.400	4
36	0.124	0.600	0.062	0.356	4

Table S2. The spontaneous mean firing rate and mean bursting rate before and after treatment with 100 μM Ni^{2+}.

No.	Mean firing rate - control (spikes/s)	Mean bursting rate - control (bursts/min)	Mean firing rate - 100 μM Ni^{2+} (spikes/s)	Mean bursting rate - 100 μM Ni^{2+} (bursts/min)	Slice No.
1	0.126	0.222	0.013	0.044	1
2	0.127	0.533	0.211	0.867	1
3	0.994	3.044	0.277	0.533	1
4	0.179	0.622	0.246	0.667	1
5	0.511	2.978	0.220	0.644	1
6	0.159	0.400	0.058	0.156	1
7	0.204	0.689	0.135	0.533	1
8	0.151	0.578	0.339	0.733	1
9	0.242	0.644	0.305	0.644	1
10	0.111	0.311	0.178	0.267	1
11	0.288	0.356	0.156	0.200	1
12	0.249	1.133	0.223	0.756	1
13	0.187	0.400	0.095	0.444	1
14	0.131	0.467	0.288	0.844	1
15	0.158	0.356	0.279	0.444	1
16	0.200	0.889	0.543	0.689	1
17	0.147	0.333	0.055	0.133	1
18	0.280	0.311	0.207	0.267	1
19	0.132	0.422	0.184	0.311	1
20	0.144	0.400	0.355	1.067	1
21	0.138	0.111	0.034	0.000	1
22	0.205	0.311	1.004	2.889	1
23	0.113	0.822	0.011	0.000	1
24	0.269	1.022	0.030	0.111	1
25	1.125	7.556	0.060	0.267	1
26	0.516	1.422	0.336	1.089	1
27	0.346	1.200	0.003	0.000	2
28	0.103	0.222	0.025	0.067	2
29	0.451	0.911	0.111	0.244	2
30	1.305	5.556	0.011	0.022	2
31	1.985	7.533	0.206	0.578	2
32	0.497	3.333	0.320	0.756	2
33	0.226	0.800	0.164	0.311	2
34	0.358	1.089	0.097	0.289	3
35	0.134	0.578	0.078	0.289	3
36	0.214	0.467	0.087	0.400	3
37	2.186	15.511	0.440	1.533	4
38	0.191	0.511	0.091	0.156	4
39	0.150	0.356	0.053	0.044	4

40	0.771	1.600	0.525	1.267	4
41	0.159	0.489	0.163	0.400	4
42	0.146	0.289	0.089	0.511	4
43	3.311	7.756	0.839	6.089	4
44	0.332	0.333	0.040	0.156	4
45	0.139	0.556	0.100	0.600	4
46	0.352	2.156	0.156	0.289	4
47	1.881	11.000	2.583	17.200	4
48	0.293	0.311	0.072	0.311	4
49	0.102	0.111	0.027	0.133	4

Table S3. The spontaneous mean firing rate and mean bursting rate before and after treatment with 200 μM Ni^{2+}.

No.	Mean firing rate - control (spikes/s)	Mean bursting rate - control (bursts/min)	Mean firing rate - 200 μM Ni^{2+} (spikes/s)	Mean bursting rate - 200 μM Ni^{2+} (bursts/min)	Slice No.
1	0.187	0.356	0.461	2.333	1
2	0.200	0.222	0.078	0.222	1
3	0.121	0.200	0.464	0.422	1
4	0.147	0.222	0.211	0.333	1
5	0.134	0.311	0.108	0.200	1
6	0.311	0.533	0.436	0.733	1
7	0.907	0.933	2.454	8.867	1
8	0.215	0.111	0.965	0.333	1
9	0.239	0.778	2.310	11.933	1
10	0.107	0.711	0.883	9.089	1
11	0.883	1.244	3.100	10.711	1
12	0.631	1.956	0.670	1.600	1
13	0.217	0.067	0.469	0.444	1
14	0.101	0.200	0.045	0.156	1
15	0.106	0.356	0.264	0.289	1
16	0.269	0.467	0.841	0.889	1
17	0.127	0.533	0.511	0.489	1
18	0.609	0.756	1.754	6.533	1
19	0.110	0.156	0.314	0.200	1
20	0.511	2.756	0.008	0.000	1
21	0.243	0.244	0.101	0.244	2
22	0.452	0.778	0.163	0.267	2
23	0.579	1.644	0.549	2.311	2
24	0.177	0.400	0.113	0.422	2
25	2.925	10.889	1.610	3.844	2
26	0.811	2.844	0.369	1.289	2
27	1.854	7.200	0.839	2.467	2
28	0.125	0.444	0.158	0.311	2
29	0.107	0.378	0.149	0.378	2
30	0.178	0.422	1.897	5.978	2
31	0.571	1.622	1.424	8.622	2
32	0.440	1.822	0.325	1.022	2
33	0.105	0.444	0.211	0.578	2
34	0.147	0.778	0.494	0.956	2
35	0.166	0.600	0.136	0.689	2
36	0.693	1.378	0.499	0.867	2
37	0.103	0.600	0.176	0.600	2
38	0.210	0.600	0.167	0.844	2
39	0.149	0.556	0.014	0.067	2

40	0.112	0.667	0.094	0.422	2
41	0.210	0.844	0.161	0.600	2
42	0.173	0.822	0.079	0.267	2
43	0.230	0.467	0.090	0.378	2
44	0.149	0.511	0.094	0.289	2
45	0.186	0.689	0.349	1.644	2
46	1.197	6.422	0.010	0.000	2
47	0.258	0.644	0.136	0.489	2
48	0.210	0.800	0.190	0.644	3
49	0.309	0.911	0.211	0.533	3
50	0.116	0.222	0.071	0.089	3
51	0.139	0.489	0.133	0.533	3
52	0.116	0.467	0.227	0.644	3
53	0.186	0.889	0.282	1.244	3
54	0.124	0.489	0.228	1.000	3
55	1.075	2.933	0.366	0.644	3
56	0.257	1.000	0.560	0.933	3
57	0.456	1.689	0.325	0.689	3
58	0.199	0.711	0.317	0.644	3
59	0.487	1.333	0.181	0.733	3
60	0.240	0.689	0.106	0.378	3
61	0.109	0.378	0.079	0.267	3
62	1.453	6.578	0.339	0.644	3
63	0.144	0.311	0.119	0.467	3
64	0.142	0.511	0.287	0.822	3
65	0.299	1.022	0.521	0.533	3
66	0.107	0.311	0.061	0.244	3
67	0.116	0.511	0.140	0.422	3
68	0.110	0.222	0.123	0.178	3
69	0.222	0.622	0.239	0.667	3
70	0.179	0.578	0.184	0.578	3
71	0.138	0.311	0.241	0.511	3
72	0.127	0.333	0.251	0.756	3
73	0.102	0.267	0.400	1.911	3
74	0.116	0.356	0.246	0.244	4
75	0.306	0.489	1.125	1.222	4
76	0.354	1.067	0.354	1.178	4
77	0.154	0.556	0.061	0.200	4
78	0.347	1.089	0.191	0.844	4
79	1.244	3.711	0.614	1.311	4
80	0.174	0.356	0.162	0.578	4
81	0.602	1.311	0.092	0.267	4
82	0.602	2.200	0.157	0.333	4
83	5.251	28.822	7.700	22.378	4

84	0.856	2.800	0.249	0.844	4
85	0.412	1.467	0.022	0.044	4
86	0.113	0.378	0.077	0.267	4
87	0.974	3.711	0.360	0.844	4
88	0.241	0.178	0.082	0.267	4
89	0.223	0.200	0.166	0.200	4
90	0.426	1.289	0.423	0.489	4

Table S4. The spontaneous mean firing rate and mean bursting rate before and after treatment with 500 μM Ni^{2+}.

No.	Mean firing rate - control (spikes/s)	Mean bursting rate - control (bursts/min)	Mean firing rate - 500 μM Ni^{2+} (spikes/s)	Mean bursting rate - 500 μM Ni^{2+} (bursts/min)	Slice No.
1	3.945	35.889	1.344	10.356	1
2	0.364	1.200	0.185	0.356	1
3	0.301	0.711	0.118	0.489	1
4	0.232	1.244	0.087	0.133	1
5	0.229	0.111	0.024	0.044	1
6	0.406	1.000	0.039	0.111	1
7	0.266	1.067	0.180	0.244	1
8	0.118	0.467	0.260	0.667	1
9	0.331	1.133	0.170	0.822	1
10	0.854	3.844	0.933	2.800	1
11	0.200	0.578	0.519	1.822	1
12	0.287	1.511	0.123	0.311	1
13	0.305	0.889	0.048	0.133	1
14	0.109	0.311	0.121	0.333	1
15	0.127	0.511	0.126	0.289	1
16	0.110	0.222	0.293	0.444	1
17	0.233	0.822	0.238	0.400	1
18	0.307	0.244	0.322	0.400	1
19	0.114	0.622	0.320	3.311	1
20	0.177	0.533	0.383	1.489	1
21	0.124	0.533	0.436	0.711	2
22	0.101	0.400	0.431	1.978	2
23	0.471	0.844	0.297	0.467	2
24	0.140	0.867	0.238	0.844	2
25	0.139	0.222	0.058	0.244	2
26	0.180	0.844	0.608	1.889	2
27	0.207	0.400	0.113	0.289	2
28	0.268	0.600	0.092	0.222	3
29	0.499	2.067	0.374	1.422	3
30	0.330	0.222	0.930	0.333	3
31	0.344	0.111	0.675	0.422	3
32	0.521	0.378	0.643	1.178	3
33	0.135	0.689	0.086	0.444	3
34	0.140	0.533	0.095	0.311	3
35	0.324	0.422	0.271	0.356	3
36	0.217	0.489	0.259	1.311	3
37	0.159	1.000	0.323	2.178	3
38	0.108	0.311	0.232	0.400	3

Table S5. The amplitude and timing of the first peak in the peri-stimulus firing rate within 50 ms post-stimulus for control and Ni^{2+}-free experimental groups.

No.	The first peak amplitude - control (Hz)	The first peak timing - control (ms)	The first peak amplitude - 0 μM Ni^{2+} (Hz)	The first peak timing - 0 μM Ni^{2+} (ms)	Slice No.
1	97.23	4.00	127.41	4.10	1
2	147.68	4.40	177.18	4.50	1
3	204.56	4.50	145.20	5.10	1
4	191.99	4.30	179.30	4.50	1
5	98.42	5.00	129.69	4.80	1
6	127.42	5.90	135.52	5.70	1
7	156.87	5.20	218.55	4.90	1
8	178.57	5.20	163.31	5.80	1
9	184.07	5.00	177.46	5.60	1
10	143.22	5.00	116.06	5.00	1
11	131.36	5.00	154.78	4.80	1
12	93.09	5.90	102.42	5.80	1
13	150.87	5.30	147.55	5.10	1
14	206.51	4.90	253.15	4.90	1
15	252.02	4.80	230.83	4.80	1
16	158.48	4.60	146.86	4.40	1
17	173.11	5.10	173.59	5.10	1
18	113.19	5.00	96.75	4.80	1
19	214.79	8.30	229.57	9.60	2
20	20.62	5.80	0.00		2
21	84.54	5.20	123.64	4.60	2
22	122.59	4.60	84.05	3.80	2
23	148.09	4.90	133.56	4.90	2
24	270.97	14.10	211.09	6.60	2
25	84.24	8.30	53.01	7.10	2
26	109.59	13.60	74.24	15.60	2
27	185.14	5.30	142.90	4.60	2
28	130.34	5.00	0.00		2
29	174.47	10.50	197.00	7.20	2
30	308.40	15.10	328.26	13.20	2
31	156.95	10.90	85.00	9.70	2
32	103.42	5.80	68.24	5.10	2
33	125.58	5.40	71.85	6.00	2
34	170.88	9.80	202.56	9.40	2
35	354.41	9.20	311.65	8.90	2
36	17.40	7.90	4.43	9.40	2
37	61.91	9.30	78.55	8.40	2
38	253.87	15.10	286.92	11.10	2
39	65.84	7.90	0.00		2

40	49.63	5.00	67.39	4.70	3
41	210.88	4.90	205.70	5.10	3
42	190.35	5.30	187.10	5.00	3
43	190.59	5.60	203.74	6.80	3
44	53.40	8.20	49.83	10.30	3
45	113.94	4.70	118.96	5.60	3
46	168.66	4.90	158.01	5.10	3
47	197.56	12.60	230.78	11.40	3
48	83.67	4.70	131.65	4.40	3
49	126.07	5.90	130.04	5.40	3
50	194.05	12.30	154.86	13.50	3
51	32.84	5.30	21.30	4.30	3

Table S6. The amplitude and timing of the first peak in the peri-stimulus firing rate within 50 ms post-stimulus for control groups and experimental groups treated with 50 μM Ni^{2+}.

No.	The first peak amplitude - control (Hz)	The first peak timing - control (ms)	The first peak amplitude - 50 μM Ni^{2+} (Hz)	The first peak timing - 50 μM Ni^{2+} (ms)	Slice No.
1	86.08	5.80	0.00		1
2	59.61	12.90	74.69	13.00	1
3	163.96	5.30	143.25	5.20	1
4	189.90	5.40	135.62	5.30	1
5	48.23	4.80	0.00		1
6	112.45	6.10	79.79	14.30	1
7	171.22	5.00	165.84	4.90	1
8	160.73	6.40	71.14	5.80	1
9	57.58	14.40	0.00		1
10	156.55	6.20	149.34	7.30	1
11	196.44	6.80	177.80	4.80	1
12	60.92	6.10	0.00		1
13	29.98	5.50	0.00		1
14	132.50	4.90	116.41	4.90	2
15	167.58	4.90	155.11	5.20	2
16	171.28	4.30	179.71	4.70	2
17	73.55	17.80	0.00		2
18	133.23	4.10	130.76	4.20	2
19	132.79	4.30	110.52	4.70	2
20	148.30	4.90	163.20	4.70	2
21	231.22	4.90	204.93	4.80	2
22	200.79	4.60	115.59	5.00	2
23	170.15	13.50	74.34	5.70	2
24	130.73	4.80	0.00		2
25	139.64	4.50	0.00		2
26	208.49	4.60	146.74	4.40	2
27	211.61	4.50	184.59	4.40	2
28	131.95	4.60	109.83	4.20	2
29	77.06	4.70	98.56	3.90	2
30	57.64	13.50	0.00		2
31	52.15	4.20	39.79	8.40	3
32	56.06	9.60	65.43	5.60	3
33	135.83	4.20	185.57	5.30	3
34	27.68	7.30	98.62	9.00	3
35	233.97	6.40	302.11	11.70	3
36	297.25	6.90	313.70	7.40	3
37	211.43	6.40	267.32	6.00	3
38	198.15	6.50	227.21	6.00	3

39	104.78	6.50	144.36	5.90	3
40	142.63	5.80	225.39	5.30	3
41	134.78	5.60	162.60	5.00	3
42	103.34	6.90	193.64	5.40	3
43	267.67	6.40	291.93	16.80	3
44	280.03	6.90	298.58	6.50	3
45	177.31	6.30	278.23	6.20	3
46	138.14	5.40	186.82	5.20	3
47	91.66	5.50	199.41	4.90	3
48	61.55	7.90	104.94	7.30	3
49	159.17	9.80	216.33	9.60	3
50	281.90	11.70	361.88	8.50	3
51	270.96	6.40	288.86	6.20	3
52	100.34	6.20	150.99	6.30	3
53	49.19	7.00	291.56	24.10	3
54	52.75	12.80	115.19	9.60	3
55	71.52	4.20	26.61	7.60	3

Table S7. The amplitude and timing of the first peak in the peri-stimulus firing rate within 50 ms post-stimulus for control groups and experimental groups treated with 100 μM Ni^{2+}.

No.	The first peak amplitude - control (Hz)	The first peak timing - control (ms)	The first peak amplitude - 100 μM Ni^{2+} (Hz)	The first peak timing - 100 μM Ni^{2+} (ms)	Slice No.
1	68.13	6.80	0.00		1
2	125.19	4.50	0.00		1
3	134.07	6.20	96.21	6.00	1
4	95.79	6.80	0.00		1
5	141.21	6.10	0.00		1
6	154.37	6.00	114.16	5.20	1
7	152.02	5.60	105.27	5.20	1
8	134.22	5.80	80.82	6.10	1
9	237.50	5.70	198.83	5.30	1
10	185.32	4.80	177.07	4.80	1
11	137.73	5.00	135.52	4.80	1
12	148.01	5.50	156.03	5.20	1
13	60.66	5.60	81.85	4.70	1
14	137.08	5.50	0.00		1
15	197.63	5.60	191.50	5.10	1
16	162.31	5.10	130.58	4.90	1
17	185.44	5.40	171.61	5.20	1
18	133.56	6.00	118.79	5.70	1
19	118.98	4.70	138.55	4.50	1
20	84.14	4.00	99.53	4.00	2
21	69.36	5.70	0.00		2
22	90.17	5.30	0.00		2
23	133.23	5.60	69.16	5.40	2
24	200.31	5.10	143.40	5.30	2
25	60.61	6.10	0.00		2
26	84.84	7.20	59.20	7.50	2
27	79.69	6.50	0.00		2
28	245.74	6.60	207.91	6.40	2
29	247.93	5.80	239.28	5.90	2
30	219.86	5.10	185.52	5.10	2
31	121.44	5.40	192.86	5.80	2
32	279.43	5.80	313.10	6.00	2
33	339.13	7.70	315.84	7.10	2
34	239.53	5.50	205.64	5.70	2
35	260.39	5.80	269.42	5.60	2
36	250.22	7.30	252.99	7.10	2
37	308.01	5.90	269.77	5.60	2
38	118.01	4.90	82.40	4.90	2

39	109.13	13.70	140.34	12.30	2
40	329.89	6.40	327.11	6.70	2
41	241.14	5.70	247.06	6.00	2
42	121.39	6.30	140.37	6.10	2
43	239.20	7.50	217.97	7.60	2
44	293.45	6.30	317.33	6.30	2
45	274.92	5.10	302.59	5.50	2
46	127.85	4.80	198.09	4.90	2
47	101.54	3.90	117.38	3.90	2
48	42.11	3.60	0.00		3
49	133.34	5.80	0.00		3
50	242.99	5.90	169.48	6.40	3
51	192.32	5.70	208.35	6.20	3
52	197.62	6.50	175.45	6.70	3
53	190.44	6.90	172.46	6.20	3
54	84.08	7.10	0.00		3
55	272.17	6.20	286.48	6.60	3
56	319.73	5.50	308.68	5.70	3
57	217.26	12.20	230.21	14.00	3
58	216.44	10.20	293.40	6.70	3
59	363.83	7.20	350.10	7.60	3
60	284.16	6.40	277.73	6.70	3
61	111.92	6.90	0.00		3
62	261.57	8.30	216.14	9.00	3
63	177.86	6.50	138.66	7.20	3
64	206.12	14.70	362.57	18.30	3
65	224.05	13.00	260.62	13.00	3
66	331.85	7.40	314.17	7.60	3
67	226.72	6.20	216.39	6.40	3
68	273.96	8.80	247.91	8.90	3
69	135.56	7.80	189.78	9.00	3
70	208.19	5.40	166.19	5.50	3
71	80.66	14.20	138.99	13.20	3
72	175.75	4.70	191.39	5.60	3
73	191.04	6.80	176.55	7.40	3
74	173.36	5.40	155.87	5.50	3

Table S8. The amplitude and timing of the first peak in the peri-stimulus firing rate within 50 ms post-stimulus for control groups and experimental groups treated with 200 μM Ni^{2+}.

No.	The first peak amplitude - control (Hz)	The first peak timing - control (ms)	The first peak amplitude - 200 μM Ni^{2+} (Hz)	The first peak timing - 200 μM Ni^{2+} (ms)	Slice No.
1	132.90	3.90	79.78	3.70	1
2	54.84	12.70	0.00		1
3	99.58	6.30	0.00		1
4	190.60	5.70	107.24	4.90	1
5	153.52	8.60	0.00		1
6	153.00	7.40	107.89	6.50	1
7	263.87	6.40	196.28	6.60	1
8	295.25	6.40	199.26	5.70	1
9	259.49	5.90	93.47	5.70	1
10	99.09	6.00	0.00		1
11	58.38	8.70	0.00		1
12	237.16	6.40	132.73	6.60	1
13	262.50	6.40	192.60	5.60	1
14	192.31	5.70	198.26	5.80	1
15	176.68	6.00	140.39	5.50	1
16	198.44	8.00	115.27	7.20	1
17	267.97	7.10	196.27	6.50	1
18	260.66	6.10	140.93	6.00	1
19	170.54	6.20	180.06	6.20	1
20	143.46	6.10	134.69	5.70	1
21	125.49	5.20	79.66	4.80	1
22	171.64	10.20	19.27	7.90	1
23	201.20	8.80	28.72	7.20	1
24	206.67	7.50	32.53	6.70	1
25	130.54	5.70	121.67	5.40	1
26	104.11	4.70	77.48	4.50	1
27	132.85	4.10	132.86	3.80	1
28	110.75	4.20	119.61	4.10	1
29	126.90	5.90	0.00		2
30	140.62	5.20	28.46	6.00	2
31	126.91	4.60	0.00		2
32	248.97	6.30	106.97	7.40	2
33	182.42	5.70	170.57	6.00	2
34	134.18	4.60	172.81	6.10	2
35	157.38	5.10	206.05	5.70	2
36	102.56	6.70	55.56	7.30	2
37	121.48	5.50	70.68	5.20	2
38	185.99	6.40	0.00		2

39	166.13	5.50	167.86	6.20	2
40	138.28	5.00	158.62	6.00	2
41	215.42	6.70	259.00	6.70	2
42	209.78	7.10	170.77	6.80	2
43	180.47	6.90	187.53	5.30	2
44	139.76	5.00	0.00		2
45	140.92	4.30	180.72	4.50	2
46	76.20	5.40	161.94	6.20	2
47	287.29	7.80	300.50	8.10	2
48	277.68	7.10	239.61	7.00	2
49	114.64	6.10	82.38	7.30	2
50	58.85	4.90	0.00		2
51	135.28	5.60	135.11	5.70	2
52	71.59	4.80	0.00		2
53	86.88	6.70	67.50	5.90	2
54	138.37	6.90	86.36	7.10	2
55	53.20	6.80	0.00		2
56	119.72	6.60	0.00		3
57	103.83	6.60	26.44	6.00	3
58	95.33	3.70	122.66	4.20	3
59	285.74	5.20	246.84	5.50	3
60	154.03	5.30	167.15	5.50	3
61	145.88	5.00	95.51	5.90	3
62	182.31	4.90	152.16	4.80	3
63	255.35	6.00	181.83	6.60	3
64	294.25	5.40	275.53	5.40	3
65	254.52	4.70	275.35	5.90	3
66	191.56	10.80	334.52	13.30	3
67	259.91	5.60	292.33	5.60	3
68	247.62	16.80	225.26	7.90	3
69	235.85	6.10	221.68	6.70	3
70	262.74	5.10	287.04	6.60	3
71	101.19	5.60	265.45	14.20	3
72	190.54	12.00	317.14	15.70	3
73	301.86	7.30	315.77	13.10	3
74	150.80	8.30	130.25	7.20	3
75	181.01	5.90	88.79	6.60	3
76	226.59	5.60	211.21	6.30	3
77	247.20	5.10	243.47	6.90	3
78	127.18	5.30	259.54	6.40	3
79	269.92	8.00	213.57	6.60	3
80	52.10	7.70	84.79	6.40	3
81	155.25	6.40	85.06	6.80	3
82	220.20	5.10	96.62	4.90	3

83	146.60	5.40	123.83	6.50	3
84	258.03	5.50	259.34	5.80	3
85	210.87	5.10	193.33	5.20	3
86	52.10	12.80	23.44	13.10	3
87	99.96	4.80	140.05	4.80	3

Table S9. The amplitude and timing of the first peak in the peri-stimulus firing rate within 50 ms post-stimulus for control groups and experimental groups treated with 500 μM Ni^{2+}.

No.	The first peak amplitude - control (Hz)	The first peak timing - control (ms)	The first peak amplitude - 500 μM Ni^{2+} (Hz)	The first peak timing - 500 μM Ni^{2+} (ms)	Slice No.
1	2.22	3.70	0.00		1
2	83.87	46.00	0.00		1
3	4.42	5.70	68.73	3.80	1
4	136.18	37.70	0.00		1
5	58.07	29.10	0.00		1
6	103.27	28.80	0.00		1
7	32.03	5.00	0.00		1
8	22.66	4.90	0.00		1
9	123.76	10.90	0.00		1
10	14.62	23.00	0.00		1
11	98.96	12.40	0.00		1
12	65.47	9.10	0.00		1
13	2.25	8.00	0.00		1
14	87.00	4.10	119.53	3.80	2
15	58.10	6.50	0.00		2
16	184.51	6.50	0.00		2
17	216.60	6.10	0.00		2
18	279.68	5.80	79.97	6.80	2
19	266.29	5.40	93.10	10.60	2
20	240.34	6.10	28.93	8.00	2
21	72.02	6.90	0.00		2
22	188.84	6.40	0.00		2
23	127.61	6.60	82.15	7.70	2
24	159.85	5.60	174.57	6.70	2
25	269.19	6.40	163.99	8.50	2
26	217.99	7.20	72.45	16.90	2
27	216.52	6.80	117.30	3.80	2
28	245.47	5.80	0.00		2
29	268.02	5.20	137.74	4.70	2
30	280.89	7.20	134.89	4.20	2
31	259.96	5.90	22.19	18.50	2
32	96.56	9.60	57.96	5.20	2
33	172.77	6.70	0.00		2
34	168.04	6.00	133.94	4.30	2
35	175.92	5.70	0.00		2
36	158.03	7.20	0.00		2
37	38.56	25.00	0.00		2
38	65.97	4.90	0.00		3

39	141.96	5.70	124.40	6.10	3
40	108.56	5.00	87.02	6.00	3
41	90.97	6.10	109.15	6.00	3
42	80.04	4.80	140.16	5.20	3
43	92.65	4.90	124.93	5.00	3
44	184.30	4.90	178.64	5.60	3
45	200.15	5.50	230.90	6.00	3
46	130.56	5.40	136.03	5.60	3
47	193.75	5.20	217.65	5.70	3
48	76.38	4.90	213.25	5.50	3
49	62.72	5.90	204.15	12.70	3
50	162.09	5.10	200.18	5.80	3
51	85.27	5.60	111.19	5.50	3
52	234.95	5.60	192.80	6.20	3
53	104.92	4.40	193.83	6.20	3
54	58.65	5.60	123.14	6.80	3
55	134.99	11.00	195.59	10.30	3
56	159.33	5.20	155.27	5.70	3
57	225.01	5.70	0.00		3
58	182.40	5.40	142.86	5.70	3
59	76.28	4.90	0.00		3
60	88.94	6.10	0.00		3
61	77.10	6.20	0.00		3
62	65.13	6.10	61.96	4.20	3
63	101.49	5.80	0.00		3
64	138.25	4.70	132.94	4.60	3
65	75.09	4.60	0.00		3

Table S10. The percentage changes in the amplitude of the first peak in the peri-stimulus firing rate within 50 ms post-stimulus induced by 100, 200, or 500 μM Ni^{2+}.

No.	The percentage changes in the amplitude of the first peak induced by Ni^{2+}		
	100 μM Ni^{2+}	200 μM Ni^{2+}	500 μM Ni^{2+}
1	-100.00	-39.97	-100.00
2	-100.00	-100.00	-100.00
3	-28.24	-100.00	-100.00
4	-100.00	-43.74	-100.00
5	-100.00	-100.00	-100.00
6	-26.05	-29.48	-100.00
7	-30.75	-25.61	-100.00
8	-39.79	-32.51	-100.00
9	-16.28	-63.98	-100.00
10	-4.45	-100.00	-100.00
11	-1.60	-100.00	-100.00
12	5.42	-44.03	-100.00
13	34.92	-26.63	37.40
14	-100.00	3.09	-100.00
15	-3.10	-20.54	-100.00
16	-19.55	-41.91	-100.00
17	-7.46	-26.76	-71.41
18	-11.06	-45.93	-65.04
19	16.45	5.58	-87.96
20	18.29	-6.11	-100.00
21	-100.00	-36.52	-100.00
22	-100.00	-88.77	-35.63
23	-48.09	-85.72	9.20
24	-28.41	-84.26	-39.08
25	-100.00	-6.80	-66.76
26	-30.22	-25.57	-45.82
27	-100.00	0.00	-100.00
28	-15.39	8.00	-48.61
29	-3.49	-100.00	-51.98
30	-15.62	-79.76	-91.46
31	58.81	-100.00	-39.97
32	12.05	-57.03	-100.00
33	-6.87	-6.50	-20.29
34	-14.15	28.79	-100.00
35	3.47	30.92	-100.00
36	1.11	-45.82	-100.00
37	-12.42	-41.82	-100.00
38	-30.18	-100.00	-12.37
39	28.60	1.04	-19.84

40	-0.84	14.70	19.99
41	2.45	20.23	75.10
42	15.64	-18.60	34.84
43	-8.88	3.91	-3.07
44	8.14	-100.00	15.36
45	10.07	28.24	4.19
46	54.94	112.50	12.33
47	15.60	4.60	179.20
48	-100.00	-13.71	225.52
49	-100.00	-28.14	23.50
50	-30.25	-100.00	30.41
51	8.34	-0.13	-17.94
52	-11.22	-100.00	84.73
53	-9.44	-22.31	109.95
54	-100.00	-37.59	44.90
55	5.26	-100.00	-2.55
56	-3.46	-100.00	-100.00
57	5.96	-74.53	-21.68
58	35.56	28.67	-100.00
59	-3.77	-13.61	-100.00
60	-2.26	8.51	-100.00
61	-100.00	-34.52	-4.86
62	-17.37	-16.54	-100.00
63	-22.04	-28.79	-3.84
64	75.90	-6.36	-100.00
65	16.32	8.18	
66	-5.33	74.63	
67	-4.55	12.47	
68	-9.51	-9.03	
69	40.00	-6.01	
70	-20.17	9.25	
71	72.32	162.33	
72	8.90	66.45	
73	-7.58	4.61	
74	-10.09	-13.62	
75		-50.95	
76		-6.79	
77		-1.51	
78		104.07	
79		-20.88	
80		62.75	
81		-45.21	
82		-56.12	
83		-15.53	

84	0.51
85	-8.32
86	-55.01
87	40.11

Table S11. Percentage change of the mean spontaneous firing rate across experimental groups relative to the control groups.

Ni^{2+} concentration	Min.	Q1	Median	Q3	Max.
50 μM	-98.22	-90.80	-55.82	-10.68	116.90
100 μM	-99.12	-75.22	-49.11	14.26	390.05
200 μM	-99.16	-51.62	-5.97	98.34	964.66
500 μM	-90.51	-51.26	0.47	115.18	328.31

Table S12. Percentage change of the mean spontaneous bursting rate across experimental groups relative to the control groups.

Ni^{2+} concentration	Min.	Q1	Median	Q3	Max.
50 μM	-99.44	-78.65	-52.48	-29.24	32.14
100 μM	-100.00	-79.18	-33.33	3.57	828.57
200 μM	-100.00	-45.04	-13.38	50.00	1434.29
500 μM	-89.29	-53.51	-21.48	104.44	432.14

Table S13. Percentage change of the amplitude of the first PFSR peak across experimental groups relative to the control groups.

Ni^{2+} concentration	Min.	Q1	Median	Q3	Max.
50 μM	-100.00	-29.62	-1.86	29.12	492.71
100 μM	-100.00	-30.19	-8.23	6.50	75.90
200 μM	-100.00	-55.01	-20.88	3.91	162.33
500 μM	-100.00	-100.00	-69.08	-2.68	225.52

Table S14. Percentage change of the amplitude of the electrical stimulation threshold of evoked activity across experimental groups relative to the control groups.

Ni^{2+} concentration	Min.	Q1	Median	Q3	Max.
50 μM	-10.00	0.00	14.29	25.00	80.00
100 μM	-35.00	0.00	20.00	33.33	100.00
200 μM	-33.33	10.61	36.67	64.39	160.00
500 μM	12.50	31.57	72.22	120.24	200.00

Table S15. The percentage of spikes in bursts of spontaneous activity for control groups and experimental groups treated with 50 μM Ni^{2+}.

No.	The % of spikes in bursts - control	The % of spikes in bursts - 50 μM Ni^{2+}	Slice No.
1	93.12	58.23	1
2	19.16	55.42	1
3	78.95	75.19	1
4	77.41	14.66	1
5	60.26	79.08	1
6	84.6	97.71	1
7	84.23	62.89	1
8	76.3	51.43	1
9	95.41	60	1
10	26.44	42.35	1
11	65.21	86.83	1
12	11.62	63.95	1
13	84.36	65.52	1
14	53.72	24.03	1
15	54.09	11.66	1
16	54.55	24.9	1
17	66.35	89.22	1
18	78.05	41.07	1
19	84.44	55.56	2
20	21.35	14.07	2
21	13.64	35.78	2
22	10.01	58.15	2
23	27.92	75.65	3
24	63.97	69.17	3
25	30.91	39.81	3
26	50.69	49.58	3
27	67.84	70.11	3
28	70.99	50	3
29	66.91	59.04	3
30	68.64	56.73	4
31	52.65	68.5	4
32	54.34	68.45	4
33	53.57	49.16	4
34	57.45	59.38	4
35	65.36	62.76	4
38	57.74	73.21	4

Table S16. The percentage of spikes in bursts of spontaneous activity for control groups and experimental groups treated with 100 μM Ni^{2+}.

No.	The % of spikes in bursts - control	The % of spikes in bursts - 100 μM Ni^{2+}	Slice No.
1	32.35	22.22	1
2	70.76	45.53	1
3	23.29	25.5	1
4	35.68	48.72	1
5	49.31	81.11	1
6	35.2	25.64	1
7	64.55	57.14	1
8	52.83	22.93	1
9	56.36	45.75	1
10	78.67	21.41	1
11	14.01	16.82	1
12	69.05	44.02	1
13	79.01	57.59	1
14	38.87	84.56	1
15	21.08	22.34	1
16	67.04	23.96	1
17	63.32	70.27	1
18	21.43	10.93	1
19	32.49	17.1	1
20	38.56	23.7	1
21	4.57	0	1
22	35.8	80.44	1
23	46.73	0	1
24	34.34	30.49	1
25	80.57	41.72	1
26	22.61	25	1
27	87.91	0	2
28	24.1	35.82	2
29	23.42	79.6	2
30	47.97	9.68	2
31	79.4	61.15	2
32	51.6	22.43	2
33	71.26	34.46	3
34	68.84	73.95	3
35	43.77	70	3
36	74.91	40.17	3
37	54.29	35.69	4
38	20.54	13.77	4
39	24.44	9.72	4
40	17.95	21.81	4

41	25.87	21.41	4
42	55.73	69.04	4
43	95.26	56.47	4
44	15.38	47.66	4
45	71.47	87.41	4
46	68.11	19.29	4
47	45.02	53.64	4
48	12.28	64.95	4
49	8.33	66.22	4

Table S17. The percentage of spikes in bursts of spontaneous activity for control groups and experimental groups treated with 200 μM Ni^{2+}.

No.	The % of spikes in bursts - control	The % of spikes in bursts - 200 μM Ni^{2+}	Slice No.
1	44.84	74.32	1
2	71.16	70.62	1
3	43.12	16.68	1
4	29.4	43.94	1
5	36.29	38.14	1
6	60.12	22.34	1
7	59.53	52.89	1
8	4.31	3.92	1
9	27.13	64.04	1
10	56.55	80.62	1
11	53.94	38.47	1
12	38.42	23.33	1
13	2.39	8.45	1
14	72.06	68.6	1
15	72.13	18.79	1
16	25.21	10.43	1
17	42.86	13.92	1
18	88.02	41.55	1
19	8.39	5.42	1
20	42.64	0	1
21	6.55	27.21	2
22	78.21	45.58	2
23	30.35	73.41	2
24	43.19	67.11	2
25	83.54	38.55	2
26	88.12	74.1	2
27	71.82	48.63	2
28	59.05	34.74	2
29	44.44	71.71	2
30	76.92	28.67	2
31	73.98	72.23	2
32	61.25	64.65	2
33	39.79	44.29	2
34	58.84	34.08	2
35	49.11	60.87	2
36	22.44	47.4	2
37	59.86	78.69	2
38	42.08	59.78	2
39	85.79	52.63	2
40	62.05	54.94	2

41	63.78	66.44	2
42	53.65	64.15	2
43	84.73	77.69	2
44	29.78	51.37	2
45	34.26	84.84	2
46	42.65	0	2
47	35.72	44.69	2
48	44.27	65.18	3
49	60.62	80.35	3
50	62.82	44.04	3
51	46.79	44.13	3
52	49.2	44.21	3
53	65.21	68.73	3
54	58.98	40.42	3
55	62.76	64.03	3
56	46.76	62.94	3
57	32.31	35.69	3
58	31.04	55.61	3
59	53.27	53.27	3
60	38.89	58.19	3
61	34.47	49.53	3
62	78.54	66.34	3
63	43.81	53.75	3
64	27.86	54.39	3
65	26.39	13.72	3
66	40.28	44.24	3
67	57.01	65.34	3
68	20.54	18.98	3
69	63.83	40.78	3
70	49.9	62.98	3
71	48.79	63.34	3
72	60.93	71.24	3
73	40.94	58.43	3
74	38.22	83.71	4
75	27.88	95.03	4
76	74.19	76.46	4
77	33.01	41.46	4
78	38.57	75.19	4
79	25.33	38.48	4
80	28.09	47.49	4
81	67.38	57.26	4
82	83.03	30.19	4
83	86.08	97.41	4
84	54.37	62.2	4

85	66.01	73.33	4
86	68.95	41.83	4
87	72.79	69.1	4
88	22.12	66.52	4
89	61.96	75.17	4
90	40.47	13.5	4

Table S18. The percentage of spikes in bursts of spontaneous activity for control groups and experimental groups treated with 500 μM Ni^{2+}.

No.	The % of spikes in bursts - control	The % of spikes in bursts - 500 μM Ni^{2+}	Slice No.
1	69.22	67.07	1
2	44.46	12.83	1
3	19.07	43.26	1
4	46.57	13.68	1
5	2.75	9.09	1
6	48.91	35.58	1
7	41.31	26.13	1
8	59.43	33.43	1
9	67.75	47.93	1
10	55.46	23.11	1
11	53.05	23.36	1
12	60.05	36.25	1
13	75.85	48.06	1
14	57.48	35.37	1
15	53.78	64.9	1
16	23.83	14.66	1
17	35.83	13.37	1
18	56.14	15.06	1
19	51.46	78.94	1
20	45.4	43.67	1
21	60.71	12.22	2
22	56.99	27.38	2
23	82.78	57.66	2
24	62.96	72.78	2
25	67.55	53.21	2
26	63.66	25.15	2
27	71.33	47.87	2
28	47.72	73.09	3
29	45.1	56.08	3
30	4.15	4.78	3
31	1.61	5.05	3
32	5.68	13.59	3
33	56.16	74.14	3
34	41.01	41.63	3
35	15.53	24.35	3
36	36.29	36.48	3
37	48.95	46.44	3
38	65.41	83.89	3
